# Activation of HIFa Pathway in Mature Osteoblasts Disrupts the Integrity of the Osteocyte/Canalicular Network

**DOI:** 10.1371/journal.pone.0121266

**Published:** 2015-03-25

**Authors:** Gui-lai Zuo, Lian-fang Zhang, Jin Qi, Hui Kang, Peng Jia, Hao Chen, Xing Shen, Lei Guo, Han-bing Zhou, Jin-shen Wang, Qi Zhou, Nian-dong Qian, Lian-fu Deng

**Affiliations:** 1 Shanghai Institute of Traumatology and Orthopaedics, Shanghai Key Laboratory for Prevention and Treatment of Bone and Joint Diseases with Integrated Chinese-Western Medicine, Ruijin Hospital, Shanghai Jiao Tong University School of Medicine, Shanghai, 200025, People's Republic of China; 2 Department of Orthopaedics, The First Affiliated Hospital of Soochow University, Suzhou, China; 3 Department of Orthopaedics, Qian Fo Shan Hospital, Shang Dong University, Ji Nan, China; Georgia Regents University, UNITED STATES

## Abstract

The hypoxia-inducible factors (HIFs), HIF-1α and HIF-2α, are the central mediators of the homeostatic response that enables cells to survive and differentiate in low-oxygen conditions. Previous studies indicated that disruption of the von Hippel-Lindau gene (*Vhl*) coincides with the activation of HIFα signaling. Here we show that inactivation of *Vhl* in mature osteoblasts/osteocytes induces their apoptosis and disrupts the cell/canalicular network. VHL-deficient (ΔVHL) mice exhibited a significantly increased cortical bone area resulting from enhanced proliferation and osteogenic differentiation of the bone marrow stromal cells (BMSCs) by inducing the expression of β-catenin in the BMSC. Our data suggest that the VHL/HIFα pathway in mature osteoblasts/osteocytes plays a critical role in the bone cell/canalicular network and that the changes of osteocyte morphology/function and cell/canalicular network may unleash the bone formation, The underlying mechanism of which was the accumulation of β-catenin in the osteoblasts/osteoprogenitors of the bone marrow.

## Introduction

Osteoblasts, which derive from bone marrow progenitors belonging to the mesenchymal lineage, form new bone at the end of the bone formation phase. Some osteoblasts die by apoptosis, some become quiescent cells lining in the bone surface, and others embedded in the bone matrix and transform into osteocytes [1,2,3]. Osteocytes make up 90–95% of all cells in the adult bone and are the longest-lived bone cells, surviving up to decades within their mineralized environment [1]. Osteocytes were once thought to be metabolically inactive cells that merely act as “placeholders” in bone or as “retired” osteoblasts [4]. However, it is now clear that osteocytes are multifunctional cells and play key regulatory roles in bone and mineral homeostasis. For example, osteocytes produce RANK ligand and sclerostin, which are key regulators of osteoclasts and osteoblasts, respectively [5,6,7]. Furthermore, osteocytes regulate bone marrow mesenchymal precursor osteogenic differentiation through WNT signaling cascade [8,9,10].

Oxygen (_O2_) is an essential metabolic substrate and serves as a regulatory signal controlling certain specific genetic programs [11]. Until now, there has been little knowledge regarding the oxygen tension in the cortical bone. If the osteocytes, which are embedded in the bone matrix, do not directly contact the blood vessels, they most likely will undergo physiological changes under the hypoxic condition in the cortical bone [4,12]. Hypoxia-inducible factor α (HIFα) is the most direct, and possibly, the only regulatory factor that plays a key role in cell differentiation and survival under hypoxic conditions [13,14,15]. Several studies showed that low _O2_ tension can promote the differentiation of osteoblasts into osteocytes *in vitro* [12,16]. To our knowledge, no in vivo studies have been performed to show the direct impact of low O2 tension or HIFs, on the maintenance of integrity of the osteocyte and canlicular network. We reported previously that VHL-deficient (ΔVHL) mice displayed significantly increased bone volume and bone vascularity, but the osteoblast proliferation and differentiation were not influenced significantly. However, in that study, the osteocyte morphology and activity were neglected [17]. Since deletion of Vhl in osteoblasts coincides with an elevation of hypoxia-inducible factor α (HIFα) expression, we sought to determine how hypoxia or HIFs influence osteocytes in order to reveal the novel functions of HIFs and explore new strategies for treating musculoskeletal injury and disease.

In this study, we conditionally inactivated VHL, a suppressor of HIFs, in mature osteoblasts/osteocytes by using the Cre recombinase driven by the osteocalcin promoter (OCN-Cre). The consequent activation of HIFα signaling in the mature osteoblasts and osteocytes of vhl-deficient mice (ΔVHL) was studied with respect to its effects on the morphology/function of osteocytes, maintenance of osteocyte viability, and changes in the cell/canalicular network. Furthermore, we investigated how the signals generated by the changes in osteocyte morphology/function and cell/canalicular network impinge on the bone marrow stromal cells (BMSCs) and attribute to the high bone mass.

## Results

### HIFα pathway plays a major role in the morphology and function of osteocytes

**Fig 1:**
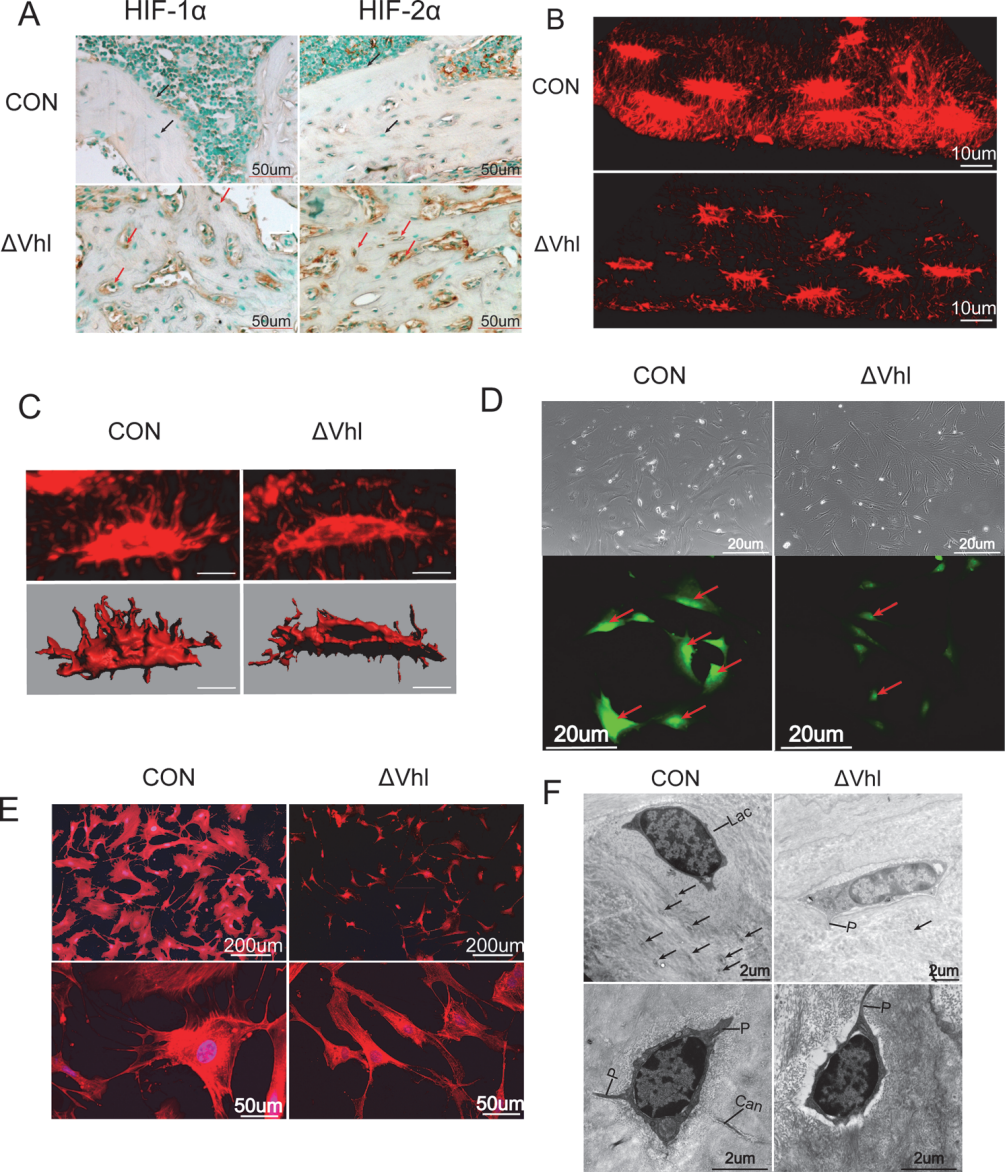
The role of HIF-α pathyway in osteocyte morphology. (A) Immunohistochemical staining indicated that there were more osteoblasts and osteocytes expressing HIF-1α and HIF-2α in ΔVHL mice than control group. (B) Representative 3D-reconstituted images of the confocal z-series slices from CON and conditional ΔVHL mice, visualized by Texas red-X-conjugated phalloidin. Bar, 10 μm. (C) Surface renderings of osteocyte cell bodies of CON and ΔVHL from the 3D-reconstituted images by IMRIS enable morphometric analyses; Bar, 10 μm. (D) Morphology of ex vivo osteocytes from the bones of CON andΔVHL mice and Immunofluorescence staining of ex vivo osteocytes with sclerostin (SOST) (E) Osteocytes from CON and conditional ΔVHL mice show stellate morphology with extensive processes in the CON. The number of dendrites is significantly decreased and less interconnected in the ΔVHL mice. (F) Osteocytes (Oc) within the lacuna (Lac) and the well-defined cell processes within the canaliculi (Can) are observed in the cross section (arrows).and section of an osteocyte intersecting three well-defined cell processes (arrows) in the CON. Osteocytes from conditional ΔVHL mice clearly display less abundant cell processes within the canaliculi (Can).

Osteocytes derive from osteoblasts. As deletion of VHL in osteoblasts results in activation of HIFs, we hypothesized that HIFs are also activated in osteocytes of VHL deficient mice. To test this we performed immunohistochemistry studies using decalcified femur samples. Results showed that in ΔVHL bone samples the numbers of HIF-1α- and HIF-2α-positive osteocytes (and osteoblasts) are increased significantly (Fig. 1A), confirming that the HIF-1α and HIF-2α are activated in the osteocytes of ΔVHL mice.

Texas red-X-conjugated phalloidin staining of decalcified femoral diaphysis showed that the osteocytes processes were largely absent inthe ΔVHL mice (6-week-old) (Fig. 1B). Morphologically, the Vhl-deficient osteocytes are significantly thinner compared to that seen in WT cells, which are spindle-shaped (Fig. 1B). The diameters of the osteocytes in the CON mouse bones ranged from 5.20 to 12.38 μm, while those in ΔVHL mice were from 3.71 to 11.80 μm (make Table 1 as Fig. 1C). Although there was no significant difference in longest diameter between CON and VHL mice, the shortest diameter of the osteocytes in the ΔVHL mice was 40% shorter than CON mice. The ratio of the longest to the shortest diameter demonstrates that the osteocytes in the ΔVHL mouse bone are thinner than those in the CON mouse bones, which makes the ΔVHL osteocytes appear longer. Both the volume and surface area of the ΔVHL osteocytes are significantly smaller than the WT osteocytes. In line with this data, we also observed a remarkable decrease (~50%) of the nuclear volume in ΔVHL osteocytes (Fig. 1C).

**Table 1:** Morphometric data of the osteocytes in cortical bone at the femoral diaphysis of ΔVhl.

	CON	ΔVhl
Longest diameter (um)	12.38±1.38	11.80±1.75
Shortest diameter (um)	5.20±0.92	3.71±0.84***
Diameter ratio,long/short	2.38	3.18
Cell surface area (um^2^)	656.03±37.2	393.51±48.03***
Cell volume (um^3^)	627.51±38.07	361.72±31.90***
Nuclear volume(um^3^)	171.55±20.5	125.02±46.09***
Volume ratio,cell/nucleus	3.66	2.89

To confirm that the morphological changes observed above are due to the deletion of VHL and activation of HIFs, we isolated osteocytes from long bones. Immunocytochemistry studies showed that the isolated cells, which displayed an osteocyte-like morphology, are positive for the osteocyte-specific marker sclerostin (SOST). It is worth noting that the fluorescence signal in WT cells is much more intense than that in ΔVHL cells, indicating that SOST expression is regulated by HIFs (Fig. 1D). Furthermore, activation of the HIFα signaling pathway (in ΔVHL mice) rendered the osteocyte cell body significantly smaller and thinner, with fewer cytoplasmic processes around. In contrast, the cytoplasmic processes were radiated in all directions and each process had many branches connecting at least two osteocytes in wild-type mice (Fig. 1E). This difference is revealed further by transmission electron microscopy (TEM) showing that the osteocytes in ΔVHL mice have less processes than that in CON mice (Fig. 1F).

**Fig 2:**
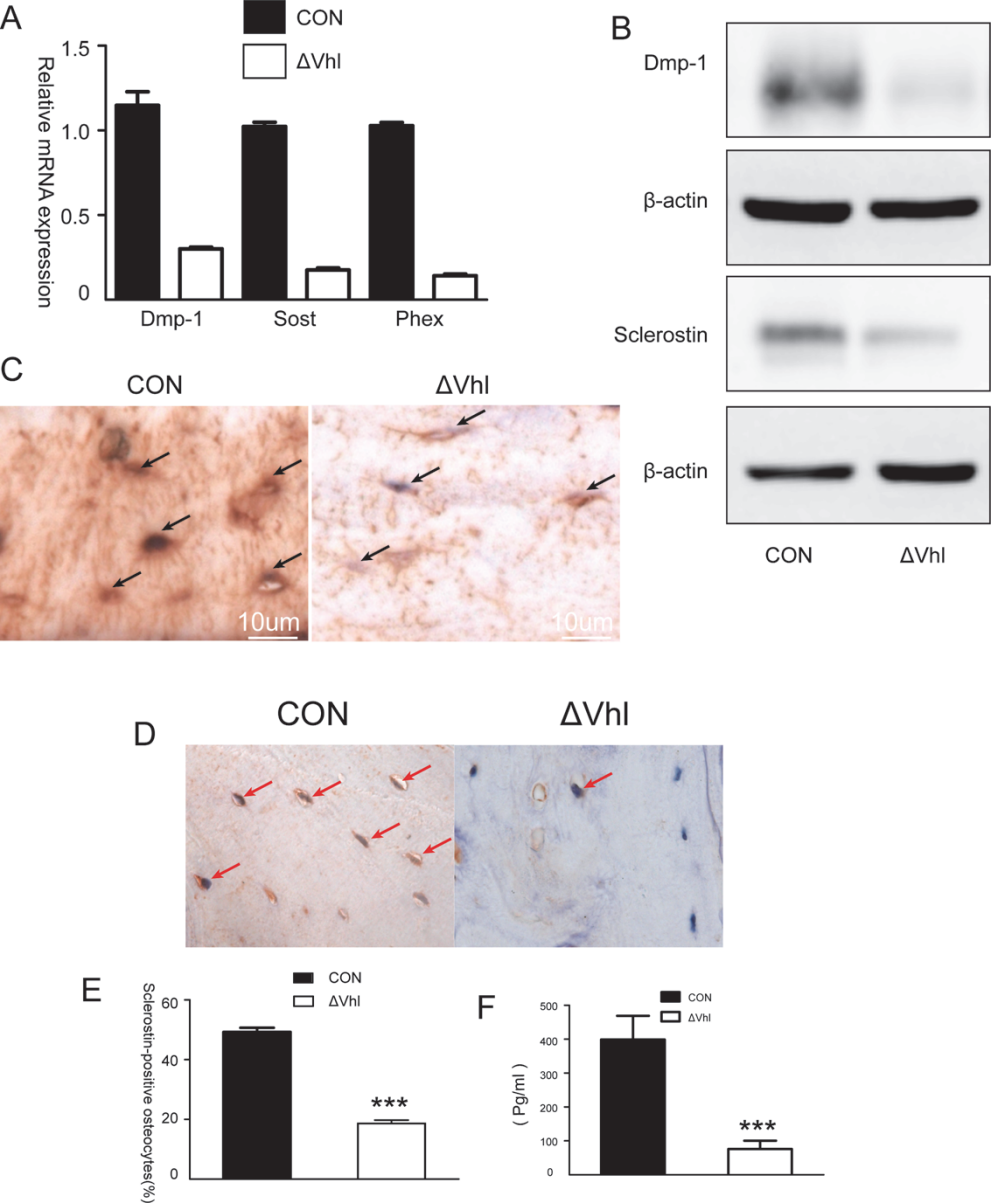
The role of HIFα pathway in the differentiation of osteocytes. (A) Quantitative PCR analysis of the differentiation markers of osteocytes. (B) Western blot analysis of DMP-1 and sclerostin proteins in the tibia of 2-month-old CON and ΔVHL mice. (C) Immunohistochemical analysis of DMP-1. Compared to the findings from CON, DMP-1 expression in the conditional ΔVHL osteocytes is dramatically reduced (arrows). (D) Immunolocalization of sclerostin in transverse sections of the mid-femoral diaphyses of 6-week-old mice. (E) The percentage of sclerostin-positive osteocytes in the mid-femoral diaphyses of the CON and ΔVHL mice (n = 6 mice for each genotype *** *p* < 0.001). (F) Serum levels of sclerostin in 3-month-old CON and ΔVHL mice (n = 9 mice for each genotype *** *p* < 0.001).

Dentin matrix protein 1 (DMP-1) and SOST are key molecules controlling osteocyte formation. To determine the effects of VHL deficiency or activation of HIFs on osteocyte development, we examined the expression of these two factors by qPCR and western blot analyses. Results showed that the expression levels of DMP-1 and SOST are decreased dramatically both at mRNA and protein levels in ΔVHL mice (4-week-old) (Fig. 2A, B). These results were further confirmed by immunohistochemistry studies, which also showed a significant decrease in the expression of DMP-1 and SOST protein in the ΔVHL bone samples (Fig. 2C, D). DMP-1 is found not only in the osteocyte cell bodies but also in the canalicular network as demonstrated by strong immunostaining in the CON mice, but not in the ΔVHL mice (Fig. 2C). The expression of SOST was detected in almost half of the osteocytes (49.27%) in the CON specimens but this number decreased significantly (18.60%) in the ΔVHL samples, indicating suppressed expression of this bone regulatory protein in ΔVHL mice (Fig. 2E). Supporting this data, serum level of SOST protein was also significantly lower in ΔVHL mice than in CON mice (Fig. 2F).

### Increase in the prevalence of osteocyte apoptosis and empty lacunae in the ΔVHL mice

**Fig 3:**
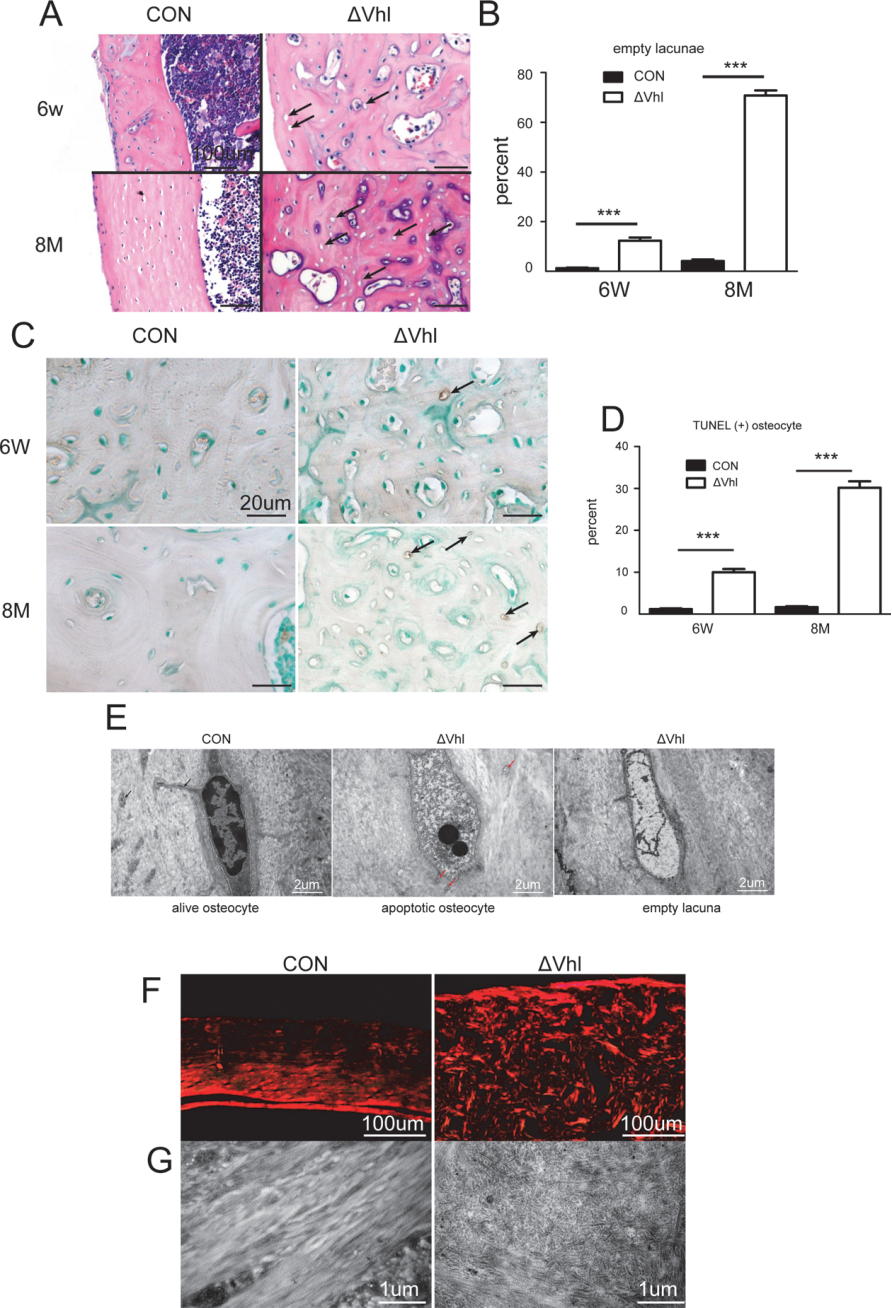
Deletion of Vhl in osteocytes and mature osteoblasts results in increased cortical osteocyte apoptosis. (A) H&E-stained sections of the cortical bone from femurs of 6-week-old and 8-month-old CON and ΔVHL mice. Empty osteocyte lacunae (arrows) can be observed in the sections obtained from the ΔVHL mice; all the osteocyte lacunae are filled with cells, as seen in the sections obtained from the CON mice. (B) Quantification of the empty osteocyte lacunae in the cortical bones of femurs from 6-week-old to 8-month-old CON and ΔVHL mice.(C) TUNEL staining of cortical bone at the diaphyses from femurs of CON and ΔVHL mice at 6 weeks and 8 months of age. (D) Frequency of TUNEL-positive lacunae. The number of TUNEL-positive lacunae was counted in mice at 6 weeks to 8 months of age, and was presented as a percentage of the total number of lacunae in the cortical bone of femurs. (E) Representative TEM images of osteocytes in the femoral midshaft. (F) Polarized microscopy of cortical bone at diaphyses of the femurs obtained from 8-month-old CON and ΔVHL mice. (G) High-power TEM images of Collagen fibrils (n = 6–9 mice for each genotype *** *p* < 0.001).

During the course of study we noted a striking difference in the numbers of empty osteocyte lacunae between ΔVHL and CON mice. The bone samples from ΔVHL mice had significantly more empty lacunae than that in CON mice (Fig. 3A). This difference became more evident as the animals age; at 6 weeks of age, the ΔVHL mice had 10 times more empty lacunae than the CON mice (10.07% vs. 1.18%) and this difference doubled at 8 months of age (70.75% vs. 4.10%) (Fig. 3B).

To determine whether the high percentage of empty lacunae in ΔVHL mice was caused by an increase in apoptosis, we performed TUNEL assays. Results showed that only about 1% of the lacunae in the cortical bone (6-week- and 8-month-old femoral diaphyses) were stained TUNEL-positive (Fig. 3C), whereas the corresponding percentage in ΔVHL mice was 10.02% at 6 weeks and 30.20% at 8 months of age (Fig. 3D). This results were further confirmed by TEM analysis which showed clear signs of apoptosis such as cytoplasmic shrinkage, chromatin condensation, and nuclear disintegration in the tibiae of the ΔVHL mice (Fig. 3E). Polarized microscopy and high-power TEM images showed normal lamellar collagen deposition in the cortical bones of CON, but the collagen fibers were disorganized in the ΔVHL mice (Fig. 3F, G), most likely due to the apoptosis of osteocytes.

### The 3D lacunocanalicular system (LCS) in the mid-diaphyses of the femurs of CON and ΔVHL mice

**Fig 4:**
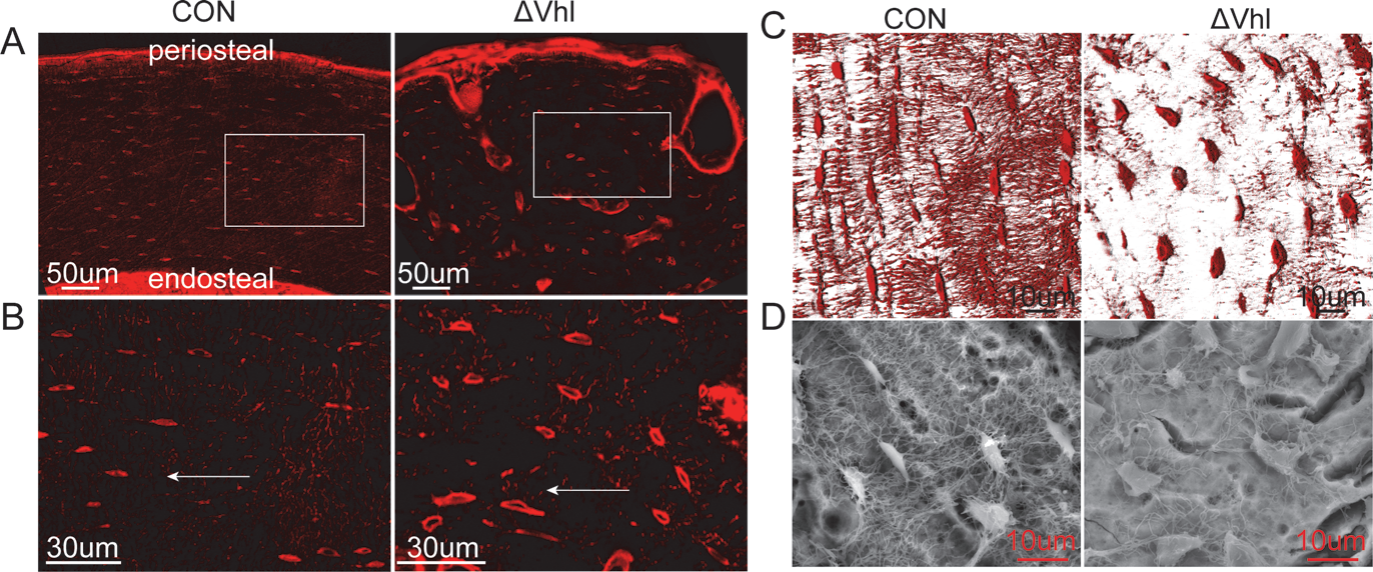
Morphological changes in the lacunocanalicular system (LCS) of ΔVHL. (A, B) The conditional ΔVHL mouse showed disorganized LCS. (arrows indicate perpendicular to the longitudinal axis of the cortical bone). (C) Basic fuchsin staining of bone tissues from CON and ΔVHL mice. (D) SEM images of the cortex of humeri in 6-week-old mice.

We next examined the LCS by staining undecalcified femoral diaphysis with basic fuchsin. In CON mice, the long axis of the lacunae is radially aligned perpendicular to the longitudinal axis of the bone and disposed parallel to each other. These long, slender canaliculi radiate in all directions around the lacuna, with the highest density perpendicular to the bone surface. In contrast, the lacunae grew in different directions and canaliculi ran randomly in the ΔVhl mice (Fig. 4A, B). In particular, the individual lacuna in ΔVHL mice has less canaliculi and an overall reduction in the density of the canalicular network connecting other lacunae and periosteal and endosteal surfaces (Fig. 4C). SEM of acid-etched, resin-casted LCS in osteocytes also showed a significant decrease in canaliculi within individual lacunae as well as reduced connectivity between individual lacunae. In the CON mice, lacunae were well-organized, uniformly shaped and distributed, whereas in the ΔVHL mice, lacunae were disorganized, decreased in number, randomly oriented, and irregularly contoured (Fig. 4D).

### VHL deficiency increases β-catenin expression in the osteoblasts/osteoprogenitors

**Fig 5:**
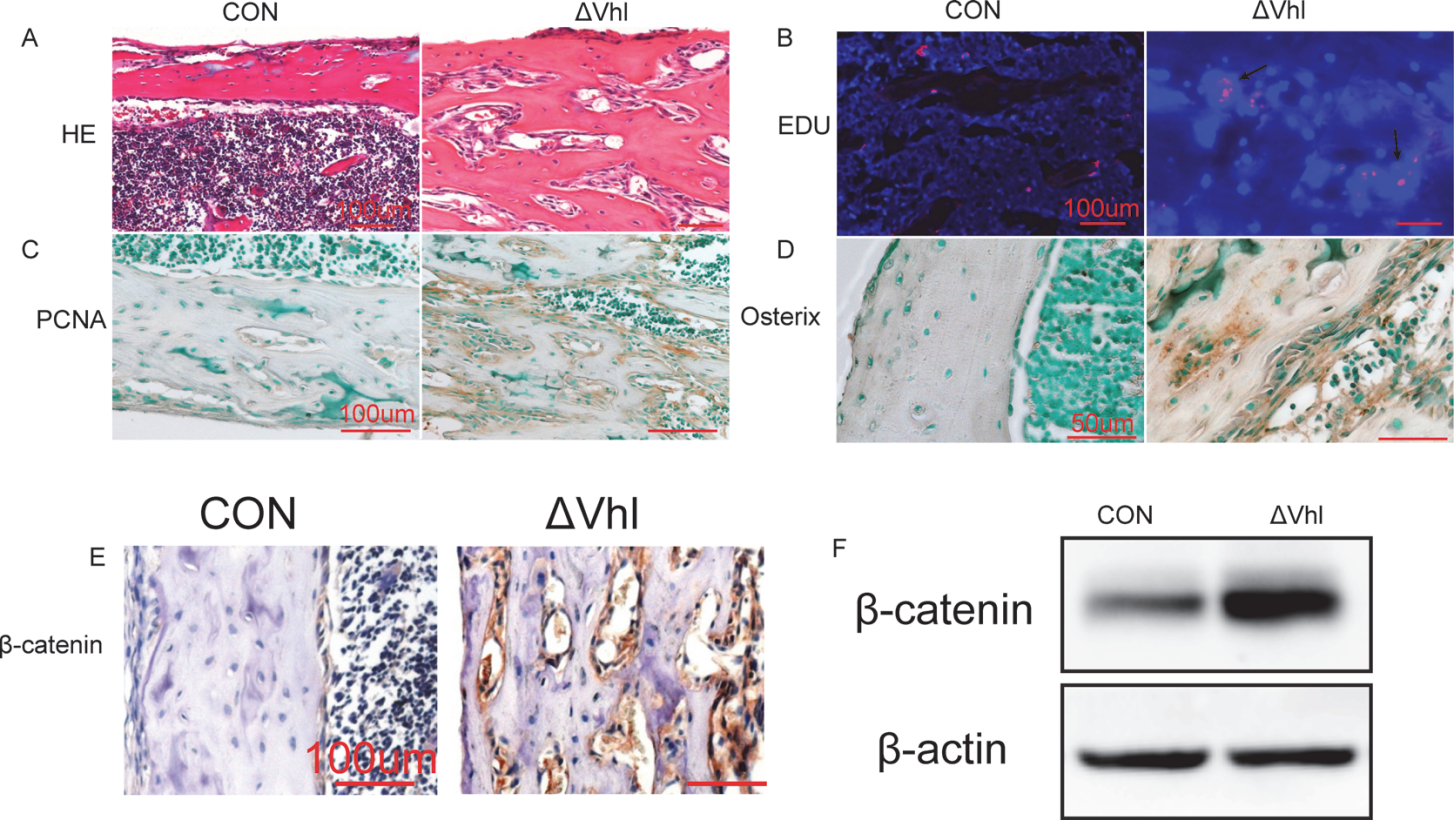
ΔVHL mice shown promoted the proliferation and differentiation of osteoblasts/osteoprogenitors and increased levels of β-catenin protein in osteoblasts/osteoprogenitors. (A) H&E staining showed diaphyseal regions of the murine femurs at 3 weeks of age. Abundant bone marrow stromal cells surround the numerous trabeculae in the ΔVHL mouse. (B) Representative images of EdU-labeled proliferating bone marrow stromal cells (red) merged with Hoechst-stained-nuclei (blue). (C) Representative histological sections of diaphyseal regions of femurs from 3-week-old ΔVHL and CON mice, after staining with anti-PCNA antibodies. (D) Immunocytochemical analysis reveals that the osterix protein is strongly detected in the abundant stromal cells in the diaphyseal regions of the femur. (E) Sections from 1-month-old CON and ΔVHL mice were IHC-stained using an antibody against β-catenin. β-Catenin-positive osteoblasts/osteoprogenitors in the diaphyseal regions of the femurs are stained brown in the ΔVHL mouse. (F) Total protein extracts were prepared from the tibiae of 1-month-old CON and ΔVHL mice and used for western blot analyses for β-catenin.

Although the bone mass increased remarkably in ΔVHL mice, their osteocyte apoptosis was also increased dramatically. Interestingly, we found that the intracortical bone in the diaphysis is surrounded by abundant stromal cells (Fig. 5A), suggesting that these cells, most likely, are osteoblast that are actively proliferating.EdU incorporation and IHC staining of PCNA results confirmed this active proliferating activity in the mesenchymal islands of the 3-week-old ΔVHL bones (Fig. 5B,C). To confirm that these proliferating stromal cells are osteoblasts, we performed immunocytochemistry studies and found that the osterix is highly expressed in these clusters in the ΔVHL mice (Fig. 5D), suggesting that these are osteoblast/osteoprogenitor cells.

Wnt/β-catenin pathway plays a key role regulating osteoblast differentiation and bone formation. To determine whether increased osteocyte apoptosis is compensated by increased osteoblast/progenitor cell proliferation in ΔVHL mice, we examined the expression of β-catenin in in 1-month-old ΔVHL mice. As shown in Fig. 5, both western blot and IHC staining results showed high levels of β-catenin expression in the bone samples of ΔVHL mice (Fig. 5E, F).

## Discussion

In this study, we showed that inactivation of VHL in osteoblasts/osteocytes results in disruption of cell/canalicular network. This disruption is attributed, in large part, to increased osteocyte apoptosis and enhanced osteoblast/progenitor cell proliferation and osteogenic differentiation, which explains why the ΔVHL mice have marked bone mass increase. The HIFα protein, which is a marker of hypoxic conditions, is less abundantly expressed in the osteocytes, whereas ablation of VHL induces the upregulation of HIFα in osteocytes. Indeed, osteocytes reside in the lacunae within the mineralized bone matrix and send forth their dendritic processes through the canaliculi to form the osteocyte lacuna-canalicular network, which connects not only to the cells on the bone surface but also to the vasculature (S1 Fig), thereby providing oxygen and nutrients to maintain cell viability in the enclosed environment [18]. Osteocytes are derived from mature osteoblasts, [4]. However, the precise mechanisms how an osteoblast becomes embedded in the bone matrix and begins its life as an osteocyte, and how osteocyte differentiation and maturation are regulated are not fully understood. A few *in vitro* studies suggested that low oxygen tension might promote the differentiation of osteoblasts into osteocytes [12,16]. In this study we provide in vivo evidence that the expression of Dmp1 and SOST, the two osteocyte specific markers, were inhibited by activation of HIFα in ΔVHL mice [18]. To our knowledge, this is the first evidence demonstrating that HIFα may regulate the differentiation and maturation of osteocytes. This finding indicates that insufficient supply of oxygen, nutrients, and survival factors, all of which can stabilize HIFα, could induce osteocyte apoptosis.

Osteocytes tend to align themselves in the direction of the principle mechanical loading [19,20]. it is thought that the orderly alignment of the osteocyte bodies can be attributed to the mechanical load on the bones [21]. Himeno-Ando et al. hypothesized that the structural differences between the osteocyte networks in the calvaria and the long bones may reflect the differences in the dynamics of the loading environment [22]. We confirm that VHL/HIFα play important roles in determining the alignment of the osteocyte cell bodies in vivo.

Osteocytes establish an extensive intracellular and extracellular communication system throughout the bone via gap junction-coupled cell processes and canaliculi. More than a decade ago, Marotti [23] proposed a model linking three types of cells of the osteogenic lineage (stromal cells, osteoblasts or bone-lining cells, and osteocytes), which says all the cells were connected through volume transmission (paracrine and autocrine stimulations) and wiring transmission (direct cytoplasmic contact). According to this model, the three cell types of the osteogenic lineage communicate from inside the bone to the bone marrow, implying that certain signals imposed on the skeletal system are spread immediately from the calcified bone to the soft bone marrow [24,25]. Targeted acute death of osteocytes by diphtheria toxin markedly reduces bone formation, demonstrating that the osteocyte network plays an important role in bone formation [26]. Mice carrying a targeted mutation in *Col1a1*, which encods a collagenase-resistant form of type I collagen, exhibit increased osteocyte apoptosis detectable at 2 weeks of age. However, excessive calvarial periosteal and tibial/femoral endosteal bone deposition was found from the ages of 3–12 months [27]. Thus, the overall function of the osteocyte network in bone formation under physiological conditions is still controversial [28] [29]. Bone formation in the cortex of the long bones was enhanced in ΔVHL mice at 3 weeks of age and increased significantly as the animals aged; this was accompanied by osteocyte deformation, irregular alignment, accumulation of empty lacunae (possibly due to osteocyte apoptosis), reduced number of osteocytes, and disruption of the osteocyte network in the entire cortical bone. We hypothesize that the osteocyte network negatively regulates bone formation and disruption of this system may enhance bone formation. Studies carried out in two mouse models support our hypothesis: BCL2 transgenic mouse, in which both the intracellular and extracellular networks were disrupted at 4 weeks of age, the bone formation was enhanced in both trabecular and cortical bones [30]. In the osteocyte-specific Gja1 knockout mice, enhanced bone formation is localized in the areas where viable osteocytes are lost [31].

**Fig 6:**
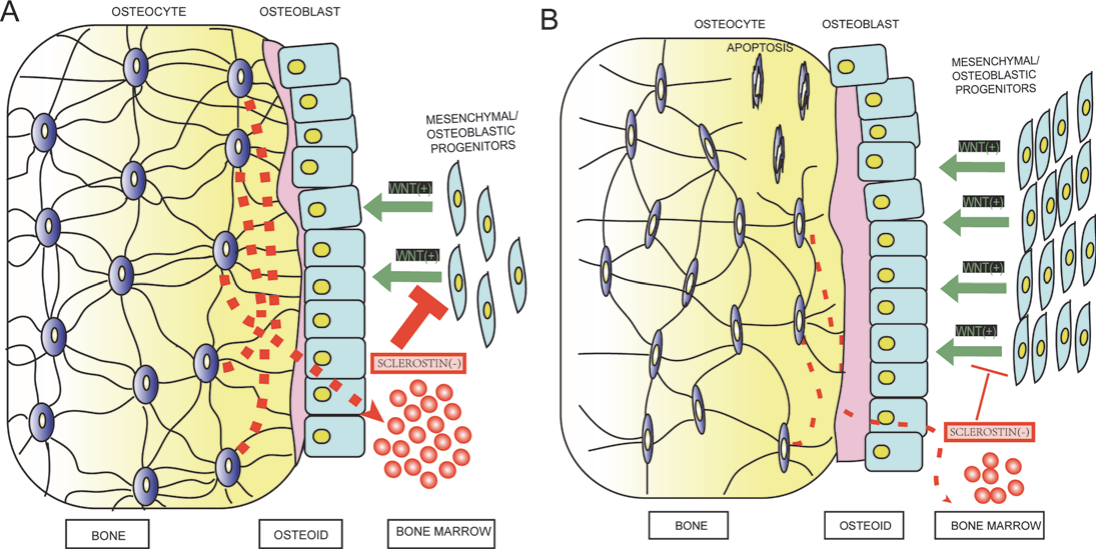
Diagram showing changes in the osteocytes after activation of the HIFα pathway and modulation of WNT/β-catenin signaling. (A) WNT/β-catenin signaling induces the proliferation of mesenchymal/osteoblastic progenitors and enhances their differentiation into the osteoblastic lineage. This process is inhibited by sclerostin secreted through the canaliculi under normal conditions. (B) After activation, the HIFα pathway reduces the sclerostin from calcified bone to the soft bone marrow, further activating WNT/β-catenin signaling.

We speculated that reduction in the number of osteocytes and their processes would enhance the proliferation of the osteoblast/osteoprogenitor lineage cells and alter their differentiation, leading to an increase in bone formation. Osteoblast knockout of Vhl had no effecton osteoclasts [17]. It is possible that signals generated by the VHL/HIFα pathway in the mature osteoblasts/osteocytes impinge on other cellular functions. A direct effect of hypoxia on osteocytes was suggested earlier by the increased migration of human mesenchymal stem cells *in vitro* [32]. Wnt/β-catenin signaling pathwayis critically involved in normal bone and cartilage formation and bone homeostasis [33].It is known that the β-catenin transgenic mice in the osteoprogenitors and/or osteoblasts results in a high bone mass phenotype characteristically very similar to our ΔVHL mice[34,35]. Sclerostin, a soluble protein produced exclusively by osteocytes in the bone, exerts its action through the thick network of osteocyte canaliculi to the bone surface and/or marrow to inhibit bone formation by inhibiting Wnt/β-catenin activation in osteogenic cells [6,36,37,38]. The activation of Wnt signaling observed in our ΔVHL mice is, most likely, due to the activation of HIFα pathway resulting in de-repression of osteocytes. More specifically, activation of HIFα resulted in (i) reduction in SOST expression in individual osteocytes, (ii) decreased prevalence of sclerostin-positive osteocytes in the total lacunae due to loss of viable osteocytes, and (iii) disruptingthe distribution of SOST due to the absence of signal transmission from the empty osteocyte lacunae to the bone surface and marrow (Fig. 6).

In conclusion, our data underscore the significant effect of VHL/HIFα pathway in mature osteoblasts/osteocytes and provide new insights into the cellular and molecular mechanisms by which osteocytes regulate bone formation and metabolism.

## Materials and Methods

### Transgenic mice

The mice protocols were approved by the Shanghai Jiaotong University Animal Study Committee and were carried out in accordance with the guide for the humane use and care of laboratory animals. Mice expressing Cre recombinase under the control of the human osteocalcin promoter (abbreviated as OC-Cre; Vhl^wild/wild^) [39] were first bred with mice in which the Vhl gene is flanked by two loxP sites (Vhl^flx/flx^) [40] (both mice kindly provided by Dr. Thomas L. Clemens, Department of Orthopaedic Surgery, Johns Hopkins University School of Medicine, Baltimore, MD) to generate OC-Cre; Vhl^flx/wild^ mice. Next, we crossed OC-Cre; Vhl^flx/wild^ with Vhl^flx/flx^ to generate OC-Cre;Vhl^flx/flx^. We then back bred OC-Cre;Vhl^flx/flx^ mice with Vhl^flx/flx^ mice to generate equal number of OC-Cre;Vhl^flx/flx^ (Vhl deficient equivalent) and Vhl^flx/flx^ (control type) littermate mice. The animals were housed 5 per cage and were maintained under a strict 12 h light: 12 h darkness cycle at 22°C with standard mice food pellets and had free access to tap water.

Genotyping was performed as described previously [17] by PCR using genomic DNA isolated from mouse tail and primers listed in S1 Table. To test the specificity of Vhl ablation,DNA was isolated from bladder, brain, calvaria, heart, humerus, intestine, liver, muscle, skin, and spleen using a DNeasy Tissue Kit (Qiagen, Germany) and amplified with Vhl Fellow and Vhl Reverse primers (S1 Table). Soft tissue and marrow were removed before DNA was isolated from calvaria and humerus. The DNA from calvaria and humerus should contain DNA from both osteoblasts and osteocytes.

### Preparation of decalcified bone tissue sections

For paraffin section, the animals were deeply anesthetized with ether and perfused through the cardiac left ventricle with 4% paraformaldehyde in 0.1Mphosphate buffer (pH 7.4). Bones were removed and immersed in the same fixation buffer at 4°C overnight. Then the bones were decalcified in daily changes of 15% EDTA in 0.01M phosphate buffer, pH 7.4, at 4°C for 1 week. After dehydration through a graded series of ethanol at 4°C, they were embedded in paraffin and sectioned at 5 um [41].

### Preparation of bone specimens for CLS analysis

The obtained femora were washed with PHEM (60 mmol/L piperazine-N’,N’-bis [2-ethane-sulfonic acid], 25 mmol/L N-[2-hydroxyethyl] piperazine-N’-[2-ethanesulfonic acid], 10 mmol/L ethylene glycol-bis [2-amino-ethyl ether]-N,N,N’,N’-tetraacetic acid, and 2 mmol/L magnesium chloride, pH 6.9). The fixation and decalcified of bone sections by the same procedure described above. The specimens were submerged in 20% sucrose to adjust their osmotic pressure, frozen in OCT compound, and sagittally sectioned into 50-μm-thick specimens. Serial cryosections of the tissue were examined under confocal laser microscope, and the resultant images were used to reconstruct and analyze 3D images [21].

### Preparation of non-decalcified bone tissue sections

Bone were immediately fixed in 70% EtOH and stored at 4°C and dehydrated in graded ethanols, and embedded in methyl-methacrylate resin following the manufacturers protocol. The EXAKT-Cutting Grinding System (EXAKT Apparatebau, Norderstedt, Germany) was used to sectioned and ground.

### Immunohistochemistry

Paraffin sections were used for immunohistochemical analysis. Briefly, sections were deparaffinized, treated with 3% H2O2 to inhibit endogenous peroxidase activity, blocked with rabbit or goat serum, and then incubated with anti-Hif-1α (Abcam,USA), anti-Hif-2α (Novus,USA), anti-Sclerostin (Santa Cruz,USA), anti-Dmp-1 (Biovision,CA) and β-catenin (Abcam, USA), at 4°C Overnight. Followed by biotinylated secondary antibodies and a peroxidase-labeled streptavidin–biotin staining technique (DAB kit, Invitrogen), nuclei were counterstained with hemalum (FARCO Chemical Supplies, Hong Kong). The slides were visualized by a microscope (ZEISS, AXIO). The slides without incubation with secondary antibody were used as negative controls.

### Osteocyte Isolation from Long Bones and immunofluorescent staining

Osteocytes were isolated from murine long bones through a process of extended collagenase digestions combined with EDTA-based decalcification. according to the report by Stern et al. [42]. Briefly, long bones from four or five mice per group were pooled together and treated as one sample,subjected to serial EDTA /collagenase digestions of 25 minutes each alternately into 10 fractions. Following each digestion, the digest solution was removed and set aside, bone pieces were rinsed with Hank’s balanced salt solution (HBSS) three times, and each rinsate was mixed with the digest solution.

Primary osteocytes isolated mice were immunologically identified using an anti-sclerostin antibody 7 days following the isolation procedure. The bone cells isolated from the bone particles at 7 days post-isolation were fixed with 4% paraformaldehyde in PBS for10 min, then incubated with PBS for 2 min. The cells were then incubated for 45 min at 25°C with gentle shaking with blocking solution: PBS + 10% goat serum. Primary antibody, sclerontin (Santa Cruz Biotechnolog, USA) at a 1:50 dilution, was applied in PBS + 3% goat serum overnight at 4°C with shaking. After washing with PBS, the secondary antibody-Alexa Fluor 488–labeled goat anti-hamster IgG (Molecular Probes, Eugene, OR, USA) at 10 μg/mL was applied for 45 min at 25°C with gentle shaking.

### Confocal Laser Scanning Microscopy

Visualization of the lacunocanalicular network: The fixed undecalcified bones were stained en bloc with basic fuchsin [43,44]. The samples were immersed in 1% basic fuchsin in methanol for 24 h; the fuchsin solution was changed every 8 h until dehydration was complete. Following the en bloc staining, the undecalcified bones were processed for embedding in methyl-methacrylate resin. The procedure on the left is known as histomorphometric analysis.

Visualization of the Osteocyte Network: In order to visualize the actin in the osteocytes, a Texas red-X-conjugated phalloidin was used following the protocol described by Sugawara, Y., et al [21]. The frozen section sections were were permeabilized by incubation in 0.5% Triton X-100 in PBS for 10 min. Specimens were then rinsed and stained for 2 days at 4°C with Texas red-X-conjugated phalloidin in PBS containing 1% BSA (1:100 dilution; excitation wavelength = 595 nm, emission wavelength = 615nm; Molecular Probes Inc., Eugene, OR). After incubation, the sections were washed with PBS, mounted in glycerin/PBS, and viewed immediately by CLSM. The 3D structure of each mouse's osteocyte network was reconstructed from CLS images of cortical femoral bone using the IMARIS software program (Bitplane AG, Zurich, Switzerland), as reported previously [21,45,46], 3–6 osteocyte was analyzed per femur for the mice (n = 6–10).

### Electron Imaging Analysis of bone

For resin-casted SEM, polished resin-casted bone samples were exposed to acid etching. The acid solution removes the bone matrix at a faster rate than the polymer that had infiltrated into the bone sample's voids (i.e., lacunae, canaliculi) during the embedding process [47,48]. The bone surface was acid etched with 9% phosphoric acid for 20 s (with the polished side upward) followed by a short rinse in deionized water (1–2 s). Subsequently, they were exposed for 5 min to 5% sodium hypochlorite and finally rinsed in deionized water. Following the acid etching procedure, the specimens were left to naturally dry at room temperature without the use of a heating cabinet. Therefore, high temperatures were avoided to ensure a mild drying process that limited the development of vapor and surface tension. The samples were then coated with gold and palladium and examined using an FEI/Quanta 250 Field-Emission Environmental Scanning Electron Microscope.

For transmission electron microscopy (TEM), Fresh femoral midshaft cross-sections obtained from CON and ΔVhl mice were fixed in 4% paraformaldehyde, 2.5% glutaraldehyde, and 0.1 M sodium cacodylate, pH 7.4, with 8.0 mM CaCl2 at 4°C overnight and then decalcified in 15% EDTA in 0.01 M phosphate buffer, pH 7.4, at 4°C for 1-week. The diaphysis was cut into small cubes, post-fixed in osmium tetroxide, dehydrated in acetone and embedded in Epon, and were examined under an H-7560 transmission electron microscope (TEM, Hitachi, Japan) [49,50].

### Quantitative Real-time PCR

Epiphyses removed and bone marrow depleted tibiae from 2-mouth CON and ΔVhl were homogenized in Trizol (Invitrogen) using a tissue homogenizer and RNA was then extracted as per the manufacturer's recommendations. Two microliters RNA was reverse transcribed using iScript cDNA Synthesis Kit (Bio-Rad), and amplified by real-time PCR using SYBR GREEN PCR Master Mix (Applied Biosystems) and primers for Dmp-1,Sost and Phex. The primers used were: Dmp-1 (Forward 5’-CTGAAGAGAGGACGGGTGATT-3’ Revers (5’-CGTGTGGTCACTATTTGCCTG-3’),Sost (Forward 5'-AGAGTA CCCCGAGCCTCCTC-3'; Reverse 5'-TCTGTCAGG AAG CGG GTG TAG-3'), Phex (Forward 5'-GTGCATCTACCAACCAGATACG-3';Reverse 5'-TCTGTTC CCCAAAAGAAAGG-3'),and β-actin (Forward 5’-TTCGTTGCCGGTCCACAC CC -3’;Reverse 5’-GCTTTGCACATGCCGGAGCC-3’).

### Western blot analysis

Western blot analysis was performed as previously described [51]. Tibias were frozen in liquid nitrogen and ground into powder using a mortar and pestle. Whole bone tissue powder were extracted by RIPA buffer on ice as described and total proteins were separated by electrophoresis on 8% SDS polyacrylamide gels. The proteins were transferred electrophoretically to nitrocellulose membranes and staining with Ponceau Red to ensure that comparable amounts of proteins were loaded and the transfer was efficient. The membranes were blocked with 5% nonfat milk in TBST for 1h at room temperature and immunobloted with Sclerostin (Santa Cruz,USA), Dmp-1 (Biovision,CA) and β-catenin (Abcam, USA) β-Actin immunoblot was used as internal control.

### In Situ End-Labeling Analysis (TUNEL)

The TUNEL method was used to detect apoptotic cells in 5-mm-thick sections of paraffin-embedded tissue using Apoptosis In Situ Detection Kit (Roch, Germany) following the manufacturer’s protocol. For negative controls, TUNEL reaction was performed without TdT enzyme.

### 5-ethynyl-2’-deoxyuridine (EdU) labeling

The nucleotide EdU was used to measure proliferation in a manner similar to that of bromodeoxyuridine (BrdU) incorporation. 6-weeks-old mice were injected with EdU (10ug/g) and sacrificed 2 h post injection. The fixation and decalcified of bone sections by the same procedure described above. EdU detection was performed using a Click-iT EdU imaging kit (Invitrogen) according to the manufacturer’s instructions. EdU-positive cells were marked by an Alexa Fluor 594 azide, imaged using a Zeiss microscope. All nuclei were stained with Hoechst dye and visualized under UV light [41].

### ELISA assay

Serum were collected from 12-month-old (n = 9–12) CON and ΔVhl mice and Sclerostin levels determined using the Mouse SOST ELISA kit (Millipore) according to the manufacturer’s protocol.

### Statistical analysis

Results were reported as the mean ± standard deviation. Unpaired Student’s t-test was used to determine statistical difference between groups. P values less than 0.05 was considered significant. (*P< 0.05; **P< 0.01; ***P<0.001).

## Supporting Information

S1 Fig Basic fuchsin staining of parietal bones of the CON and ΔVHL mice. The arrows indicate vascularization in the ΔVHL mouse.
(TIF)

S1 Table Primer sequences used for genotyping (primers should be paired). (DOC)

S1 File Raw data. (PDF)
