# Supplementary figures and images for: Activation of HIFa Pathway in Mature Osteoblasts Disrupts the Integrity of the Osteocyte/Canalicular Network
Source: PLoS One. 2015 Mar 25;10(3):e0121266. doi: 10.1371/journal.pone.0121266 (PMC4373796; doi:10.1371/journal.pone.0121266)

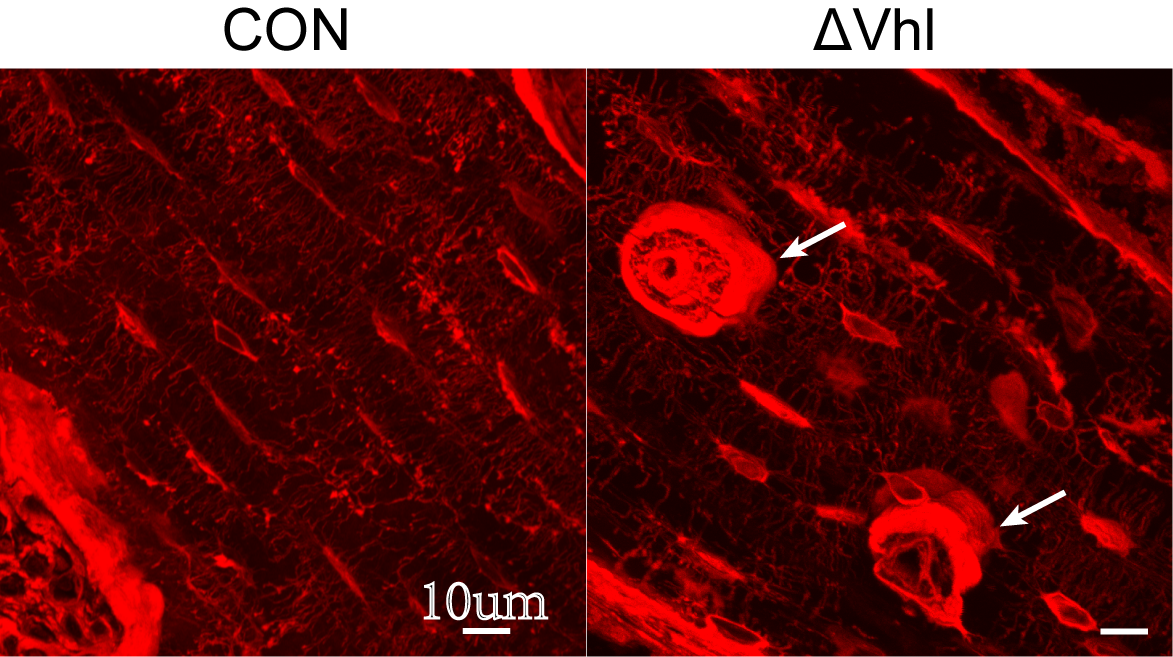

Supplement: S1 Fig — The arrows indicate vascularization in the ΔVHL mouse. (TIF) [file pone.0121266.s001.tif]
