# Supplementary material for: Activation of HIFa Pathway in Mature Osteoblasts Disrupts the Integrity of the Osteocyte/Canalicular Network
Source: PLoS One. 2015 Mar 25;10(3):e0121266. doi: 10.1371/journal.pone.0121266 (PMC4373796; doi:10.1371/journal.pone.0121266)
Supplement: S1 Table — (DOC) [file pone.0121266.s002.doc]

Supplement Table 1 Primer sequences used for genotyping (primers should be paired)

| TSH | 5’-tcc,tca,aag,atg,ctc,att,ag-3’ | 386 |
| --- | --- | --- |
|  | 5’-gta,act,cac,tca,tgc,aaa,gt-3’ |
| OST | 5’-caa,ata,gcc,ctg,gca,gat-3’ | 300 |
| RBG | 5’-tga,tac,aag,gga,cat,ctt,cc-3’ |
| VHL | 5’-cta,ggc,acc,gag,ctt,aga,ggt,ttg,cg-3’ | 450 |
|  | 5’-ctg,act,tcc,act,gat,gct,tgt,cac,ag-3 |
