# Supplementary material for: Activation of HIFa Pathway in Mature Osteoblasts Disrupts the Integrity of the Osteocyte/Canalicular Network
Source: PLoS One. 2015 Mar 25;10(3):e0121266. doi: 10.1371/journal.pone.0121266 (PMC4373796; doi:10.1371/journal.pone.0121266)
Supplement: S1 File — (PDF) [file pone.0121266.s003.pdf]

Raw data for figure 1

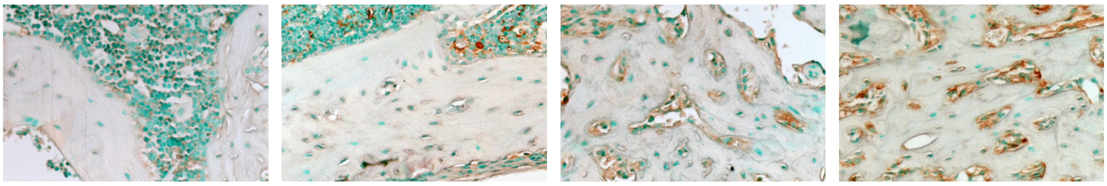

CON -HIF-1α proteins CON- HIF-2α proteins ΔVHL -HIF-1α proteins ΔVHL-HIF-2α proteins

Immunohistochemical staining for wild-type control (CON) without the HIF-1α and HIF-2α proteins present in osteoblasts and osteocytes

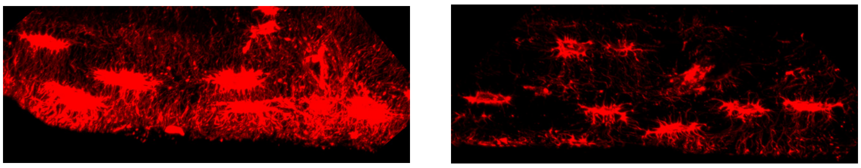

CON-Texas red-X-conjugated phalloidin ΔVHL -Texas red-X-conjugated phalloidin

Representative 3D-reconstituted images of the confocal z-series slices from wild-type control (CON) and conditional Vhl-deficient (ΔVHL) mice, visualized by Texas red-X-conjugated phalloidin.

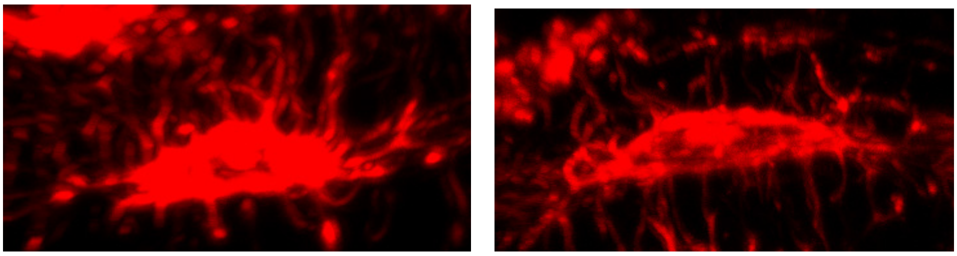

Sigle –CON-Osteocyte Sigle –ΔVHL –Osteocyte

Surface renderings of osteocyte cell bodies of CON and ΔVHL from the 3D-reconstituted images by IMRIS enable morphometric analyses;

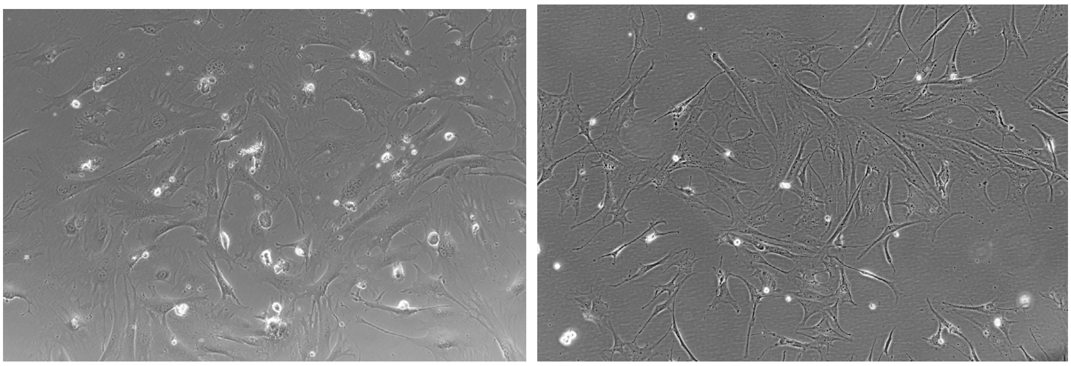

CON-ex vivo osteocytes ΔVHL-*ex vivo* osteocytes

Morphology of *ex vivo* osteocytes from the bones of wild-type control (CON) and conditional Vhl-deficient (ΔVHL) mice.

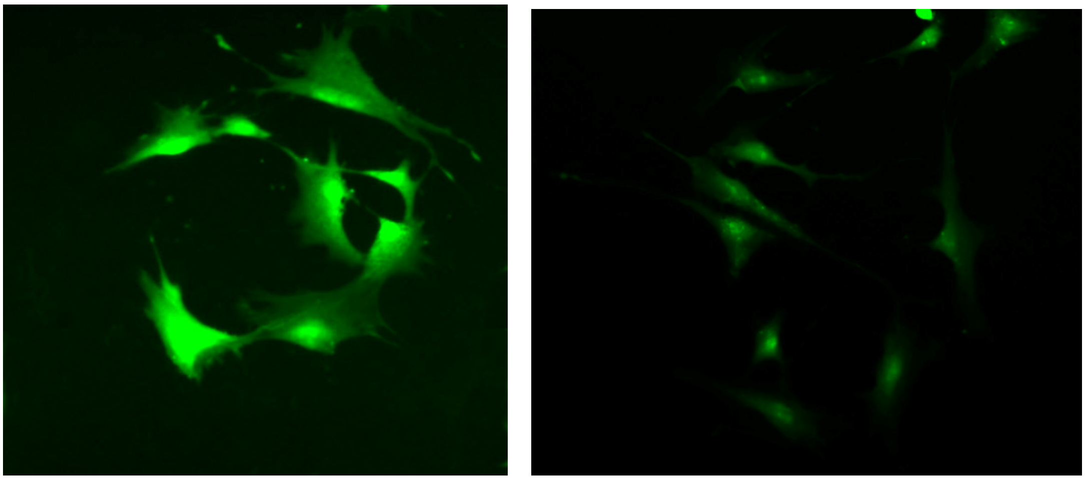

CON-ex vivo osteocytes with SOST ΔVHL -ex vivo osteocytes with SOST

Immunofluorescence staining of *ex vivo* osteocytes

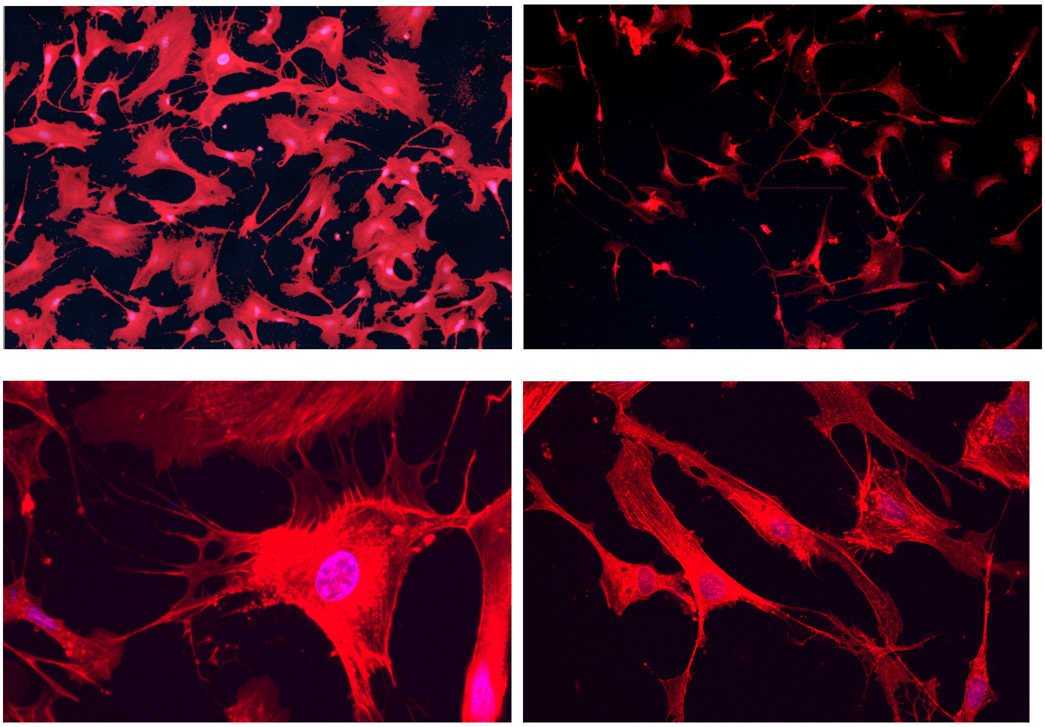

CON-ex vivo osteocytes with phalloidin ΔVHL -ex vivo osteocytes with phalloidin

Morphology of *ex vivo* osteocytes from the bones of wild-type control (CON) and conditional Vhl-deficient (ΔVHL) mice.

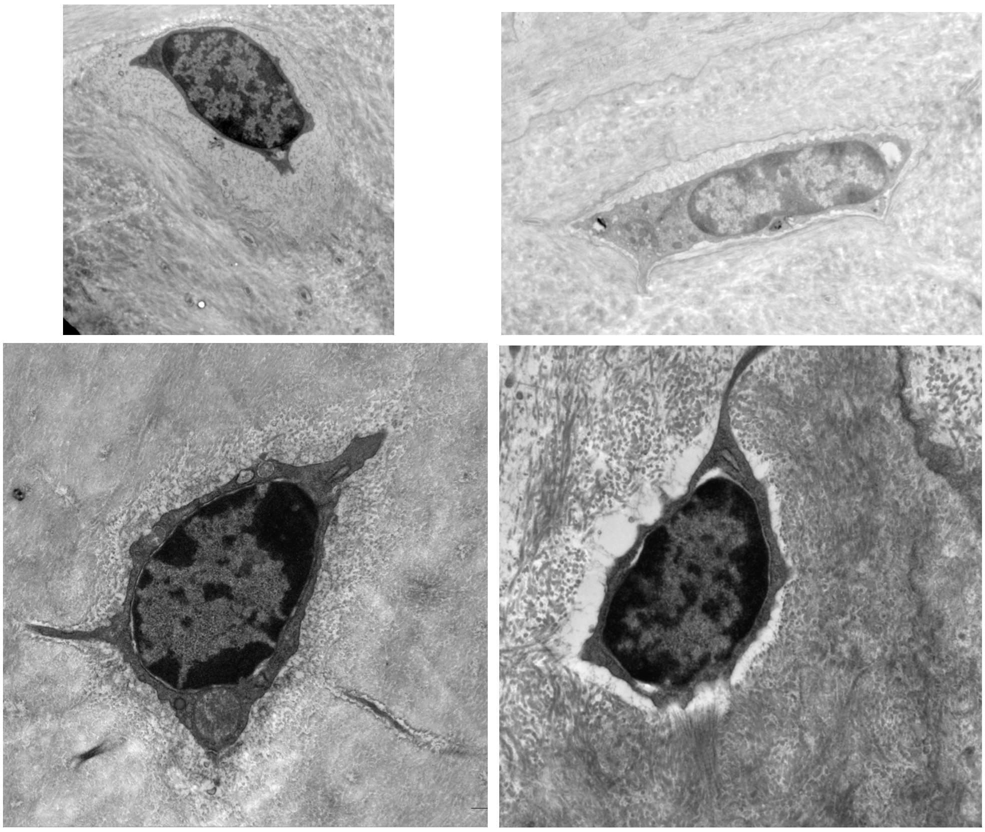

CON-Osteocyte ΔVHL –Osteocyte

Representative TEM images of osteocytes in the femoral midshaft.

Raw data for figure 2

Dmp-1		Sost		Phex	
CON-RQ	ΔVHL-RQ	CON-RQ	ΔVHL-RQ	CON-RQ	ΔVHL-RQ
1	0.3082807	1	0.1496758	1	0.1362326
1.16569	0.3136196	1.073038	0.1935637	1.061652	0.1614932
1.277888	0.2815783	0.9959073	0.1823084	1.024056	0.1286889

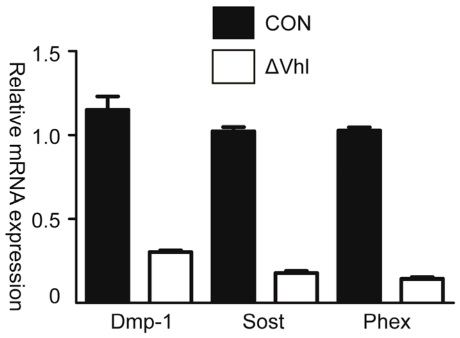

Quantitative PCR analysis of the differentiation markers of osteocytes，make Figure as Table .

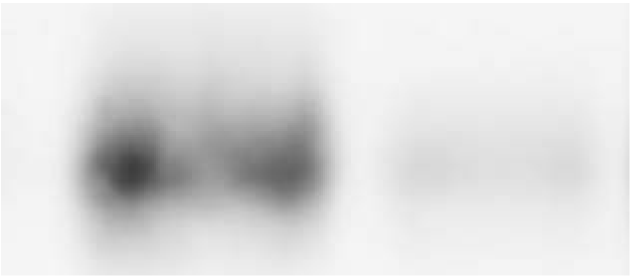

CON-dmp-1 ΔVHL-dmp-1

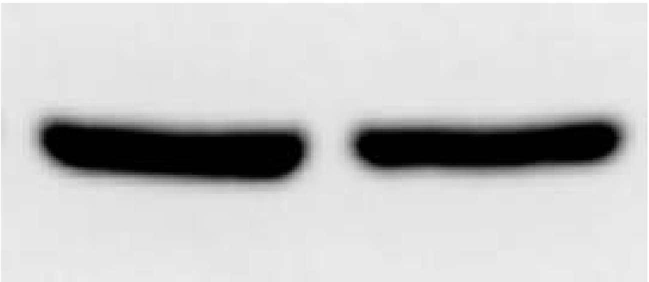

CON-DMP-1-actin ΔVHL-DMP-1-catin

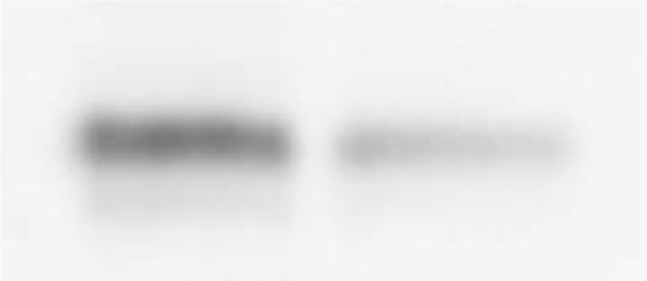

CON-SOST ΔVHL-SOST

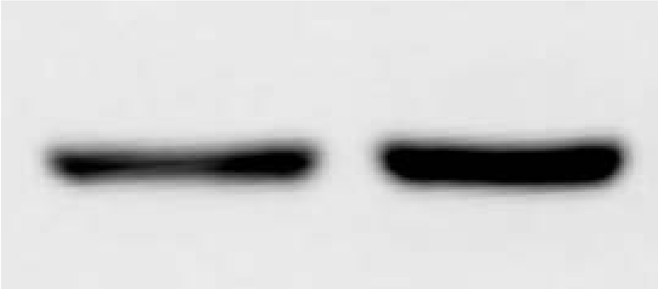

CON-SOST-actin ΔVHL-SOST-catin

Western blot analysis of DMP-1 and sclerostin proteins in the tibia of 2-month-old CON and ΔVHL mice.

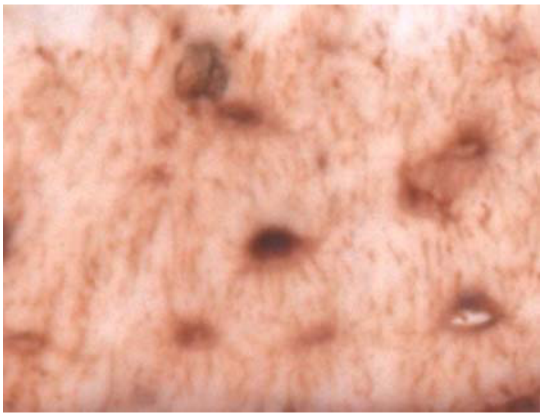

CON-Immunohistochemical analysis of DMP-1

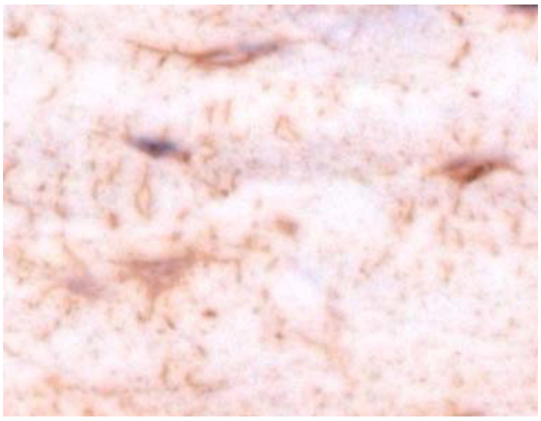

ΔVHL -Immunohistochemical analysis of DMP-1

Immunohistochemical analysis of DMP-1

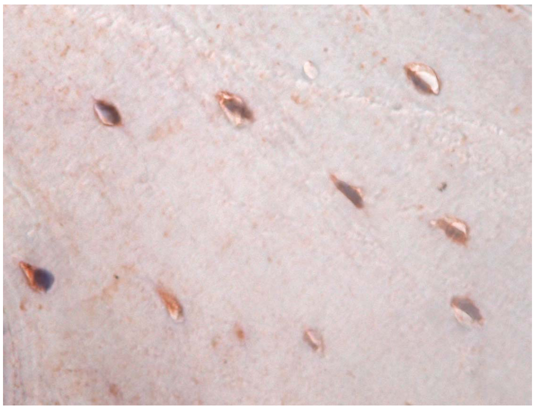

CON-Immunohistochemical analysis of SOST

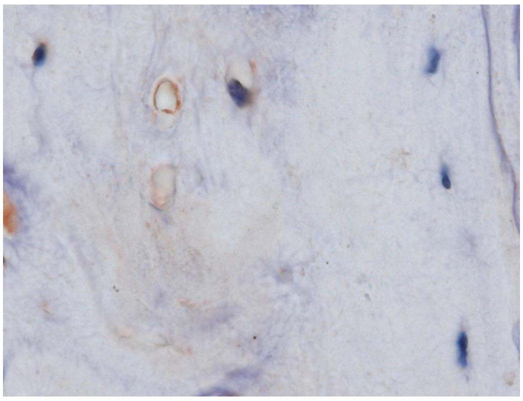

ΔVHL -Immunohistochemical analysis of SOST

Immunolocalization of sclerostin in transverse sections of the mid-femoral diaphyses of 6-week-old mice.

The percentage of sclerostin-positive osteocytes（%）	
CON-6W	ΔVHL-6W
52.5	23.42344234
53.4883721	22.0338983
45.9459459	19.0789474
52.77777778	16.9230769
46.875	13.2075
42.4242424	17.8571429
48.3870968	15.7407
51.754386	20.54

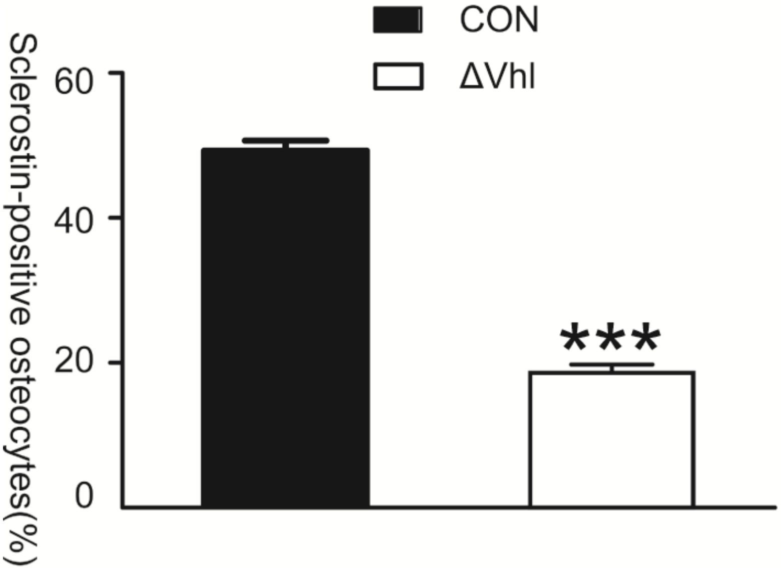

The percentage of sclerostin-positive osteocytes, make Figure as Table

Serum levels of sclerostin in 3-month-old CON and ΔVHL mice	
CON	ΔVHL
74.8484	53.31326
387.7174	49.25003
437.2889	7.805041
479.9528	9.430334
395.8439	243.8789
68.34723	85.81913
688.803	135.3906
452.3228	70.37885
605.9131	27.71489

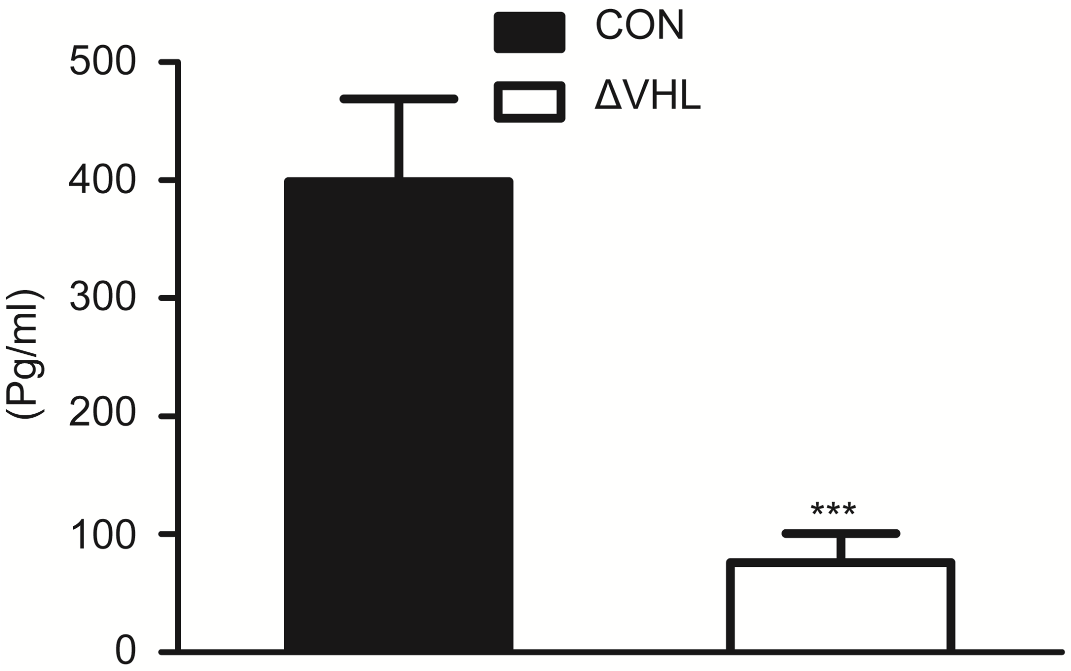

Serum levels of sclerostin in 3-month-old CON and ΔVHL mice

Serum levels of sclerostin in 3-month-old CON and ΔVHL mice, make Figure as Table

Raw data for figure 3

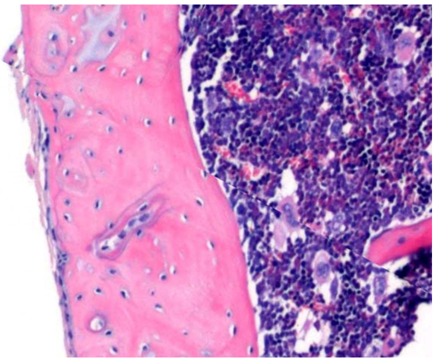

H&E-stained –CON-6W

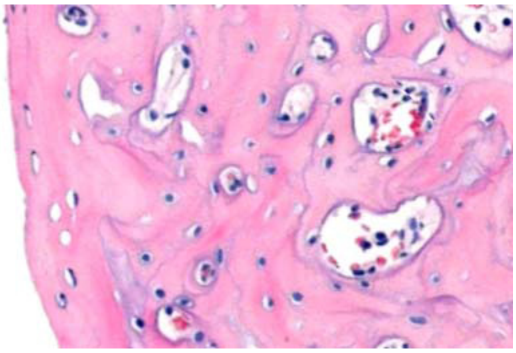

H&E-stained –ΔVHL -6W

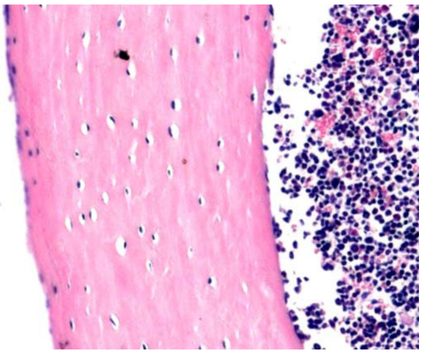

H&E-stained –CON-8M

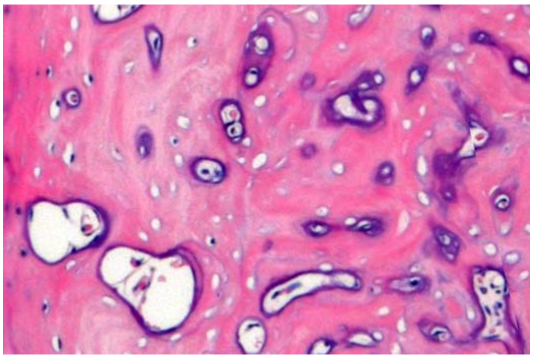

H&E-stained –ΔVHL -8M

H&E-stained sections of the cortical bone from femurs of 6-week-old and 8-month-old CON and ΔVHL mice.

Quantification of the empty osteocyte lacunae in the cortical bones of femurs			
CON-6W（%）	ΔVHL -6W（%）	CON-8M（%）	ΔVHL-8M（%）
1	13	3	71
0.8	9	3	72
1	9	6	73
0.9	9	4	70
1	10	5	62
2	15	1	73
3	17	7	64
0.6	20	4	81
2	7		
0.2	14		

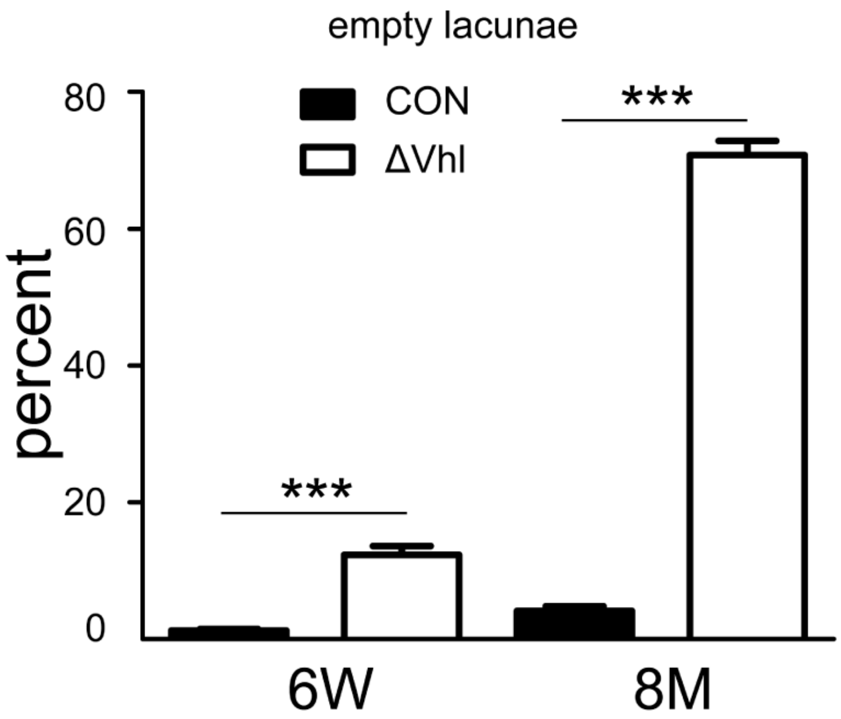

Quantification of the empty osteocyte lacunae in the cortical bones of femurs, make Figure as Table

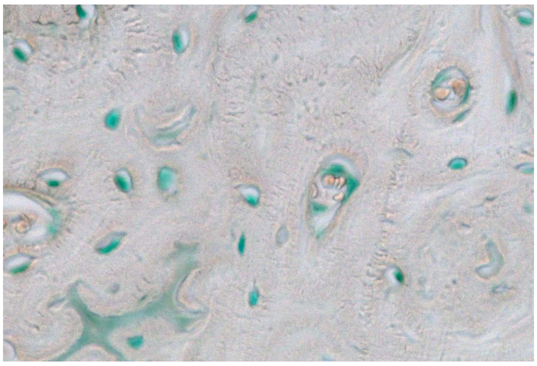

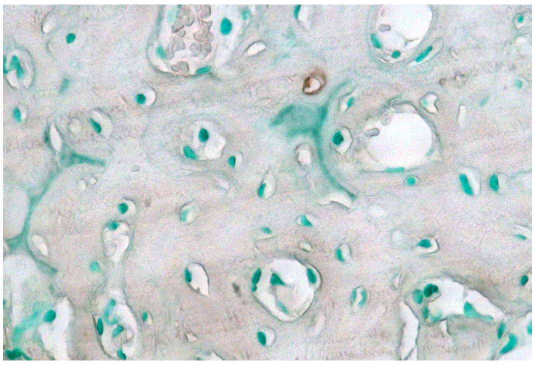

TUNEL staining-CON-6W

TUNEL staining-ΔVHL -6W

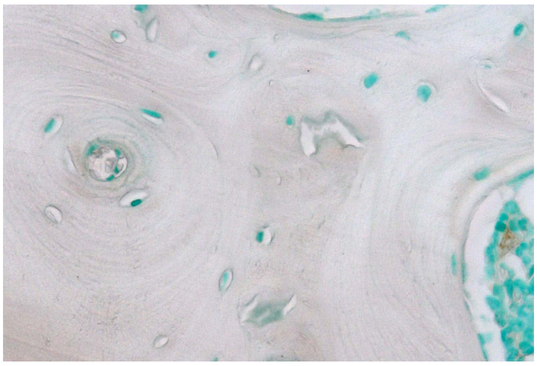

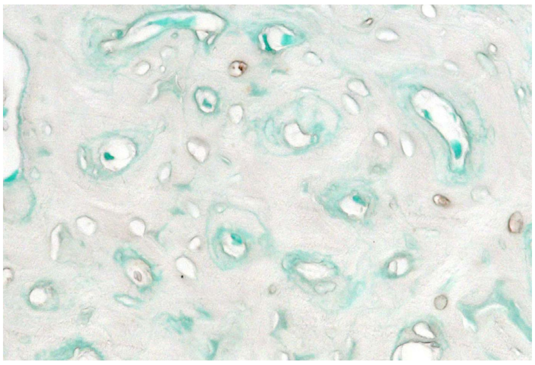

TUNEL staining-CON-8M

TUNEL staining-ΔVHL -8M

TUNEL staining of cortical bone at the diaphyses from femurs of CON and ΔVHL mice at 6 weeks and 8 months of age

Frequency of TUNEL-positive lacunae			
CON-6W	ΔVHL-6W	CON-8M	ΔVHL-8M
1.4	9.23	1.52	26.28571
0.83	8.75	1.11	31.97969
1.32	12.19	1.54	21.59091
0.62	10.32	1.2	34.23913
2.13	6.74	2.11	30.24691
1.37	11.76	2.73	34.64052
1.45	9.03	1.56	23.62205
1.32	14.11		35.50724
0.06	8.05		31.3253
1.57			32.515

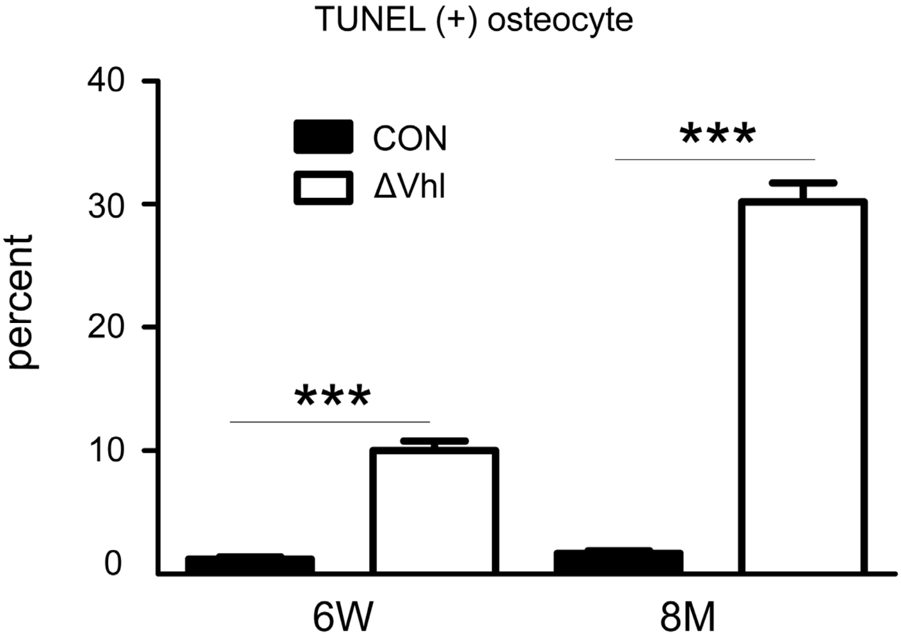

Frequency of TUNEL-positive lacunae, make Figure as Table

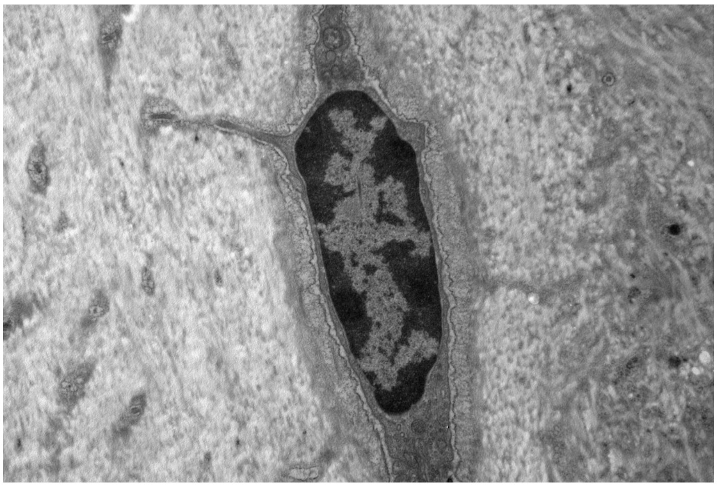

CON- TEM images of osteocytes-alive

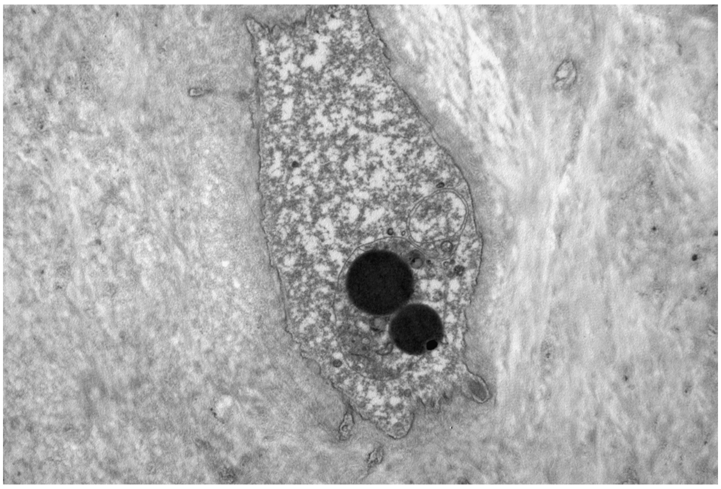

ΔVHL -TEM images of osteocytes-apoptotic osteocyte

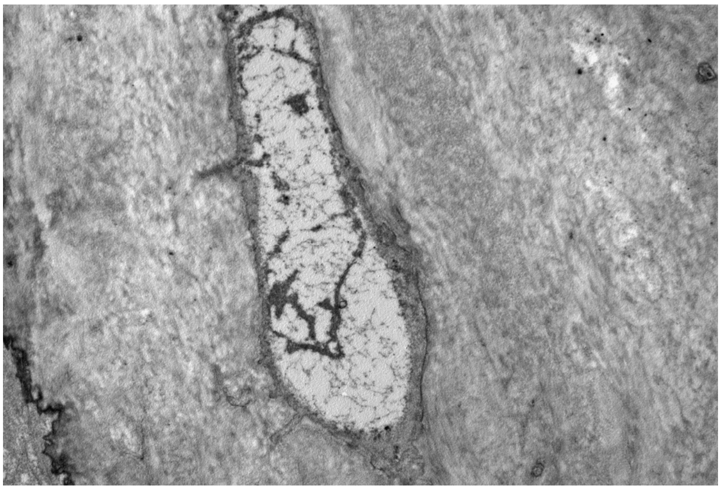

ΔVHL -TEM images of osteocytes-empty lacuna

Representative TEM images of osteocytes in the femoral midshaft.

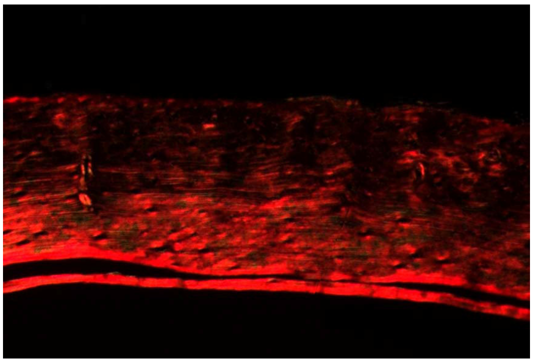

Polarized microscopy of cortical bone-CON

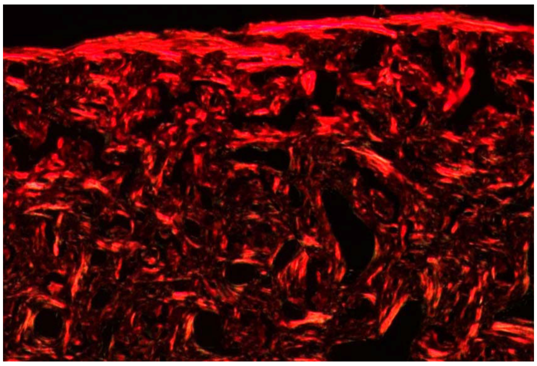

Polarized microscopy of cortical bone-ΔVHL

Polarized microscopy of cortical bone at diaphyses of the femurs obtained from 8-month-old CON and ΔVHL mice

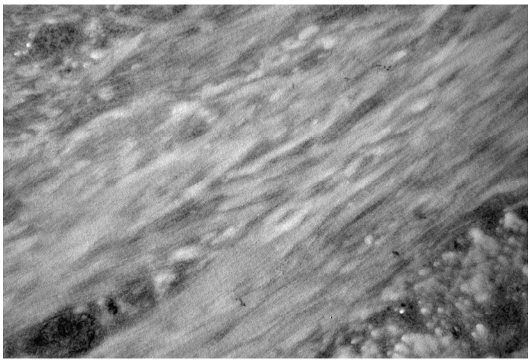

CON- High-power TEM images of Collagen fibrils

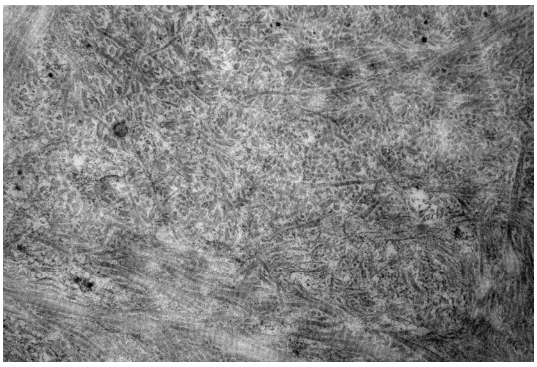

ΔVHL- High-power TEM images of Collagen fibrils

High-power TEM images of Collagen fibrils

Raw data for figure 4

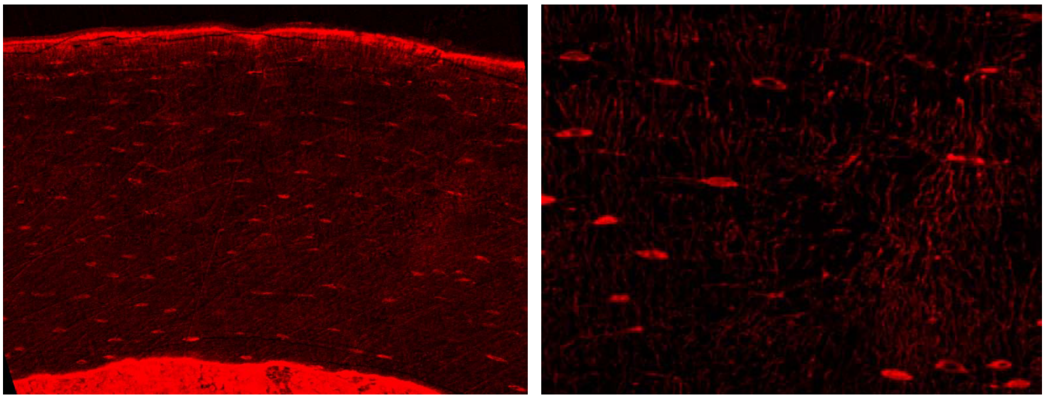

lacunocanalicular system (LCS) of CON

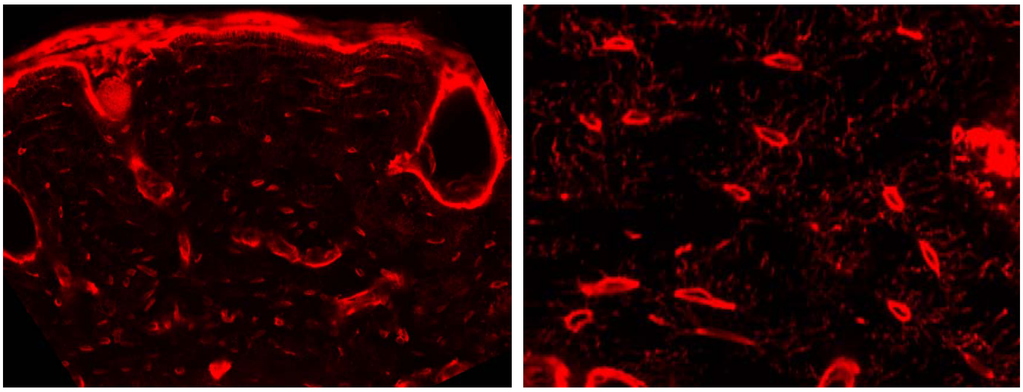

lacunocanalicular system (LCS) of ΔVHL

The conditional ΔVHL mouse showed disorganized LCS.

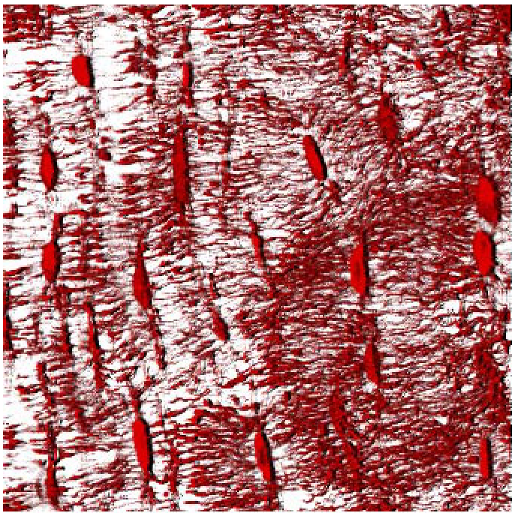

3D- lacunocanalicular system-CON

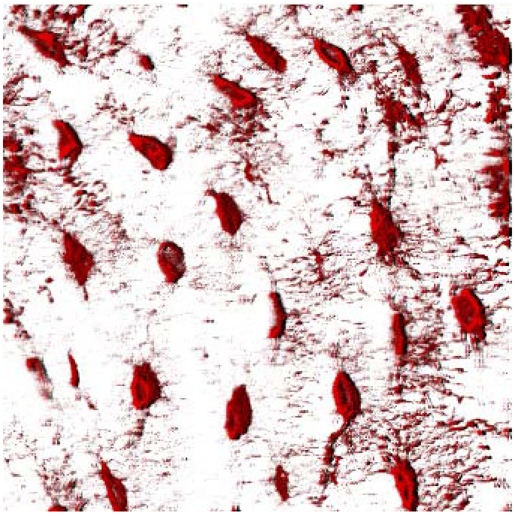

3D- lacunocanalicular system-ΔVHL

Basic fuchsin staining of bone tissues from CON and ΔVHL mice

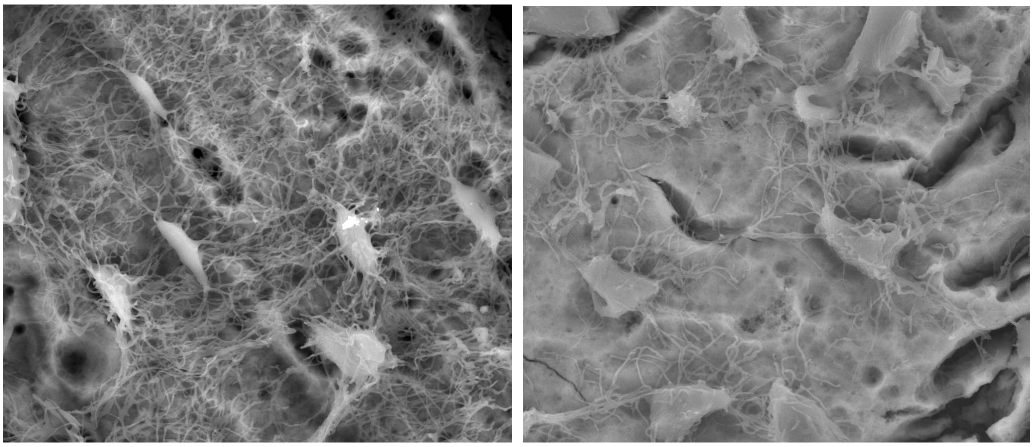

SEM images-CON SEM images-ΔVHL

SEM images of the cortex of humeri in 6-week-old mice

Raw data for figure 5

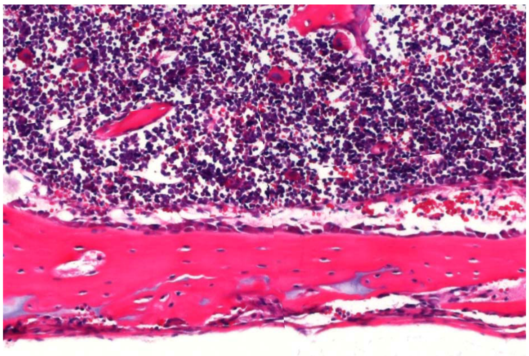

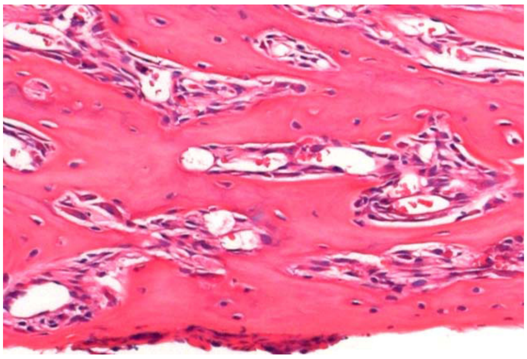

H&E staining-CON-3W

H&E staining-ΔVHL -3W

H&E staining showed diaphyseal regions of the murine femurs at 3 weeks of age. Abundant bone marrow stromal cells surround the numerous trabeculae in the ΔVHL mouse

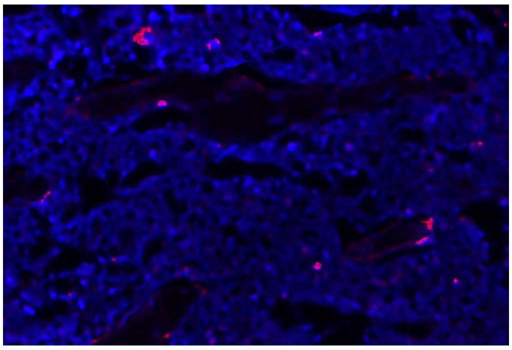

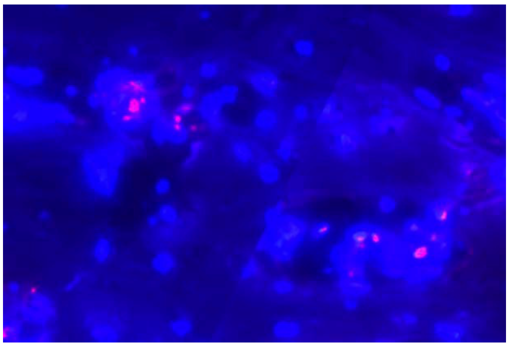

EdU-labeled proliferating cells-CON

EdU-labeled proliferating cells-ΔVHL

Representative images of EdU-labeled proliferating bone marrow stromal cells (red) merged with Hoechst-stained-nuclei (blue).

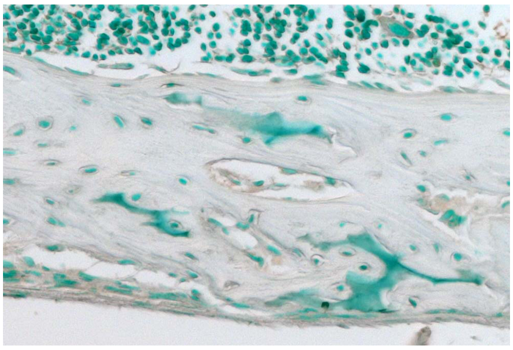

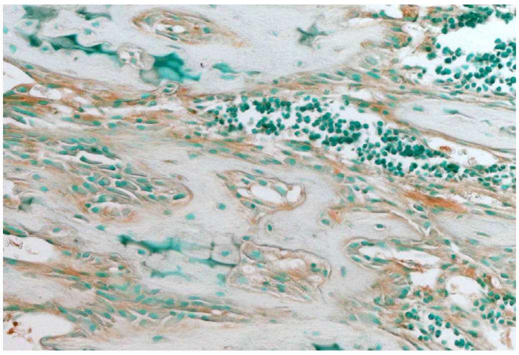

staining with anti-PCNA-CON

staining with anti-PCNA-ΔVHL

Representative histological sections of diaphyseal regions of femurs from 3-week-old ΔVHL and CON mice, after staining with anti-PCNA antibodies.

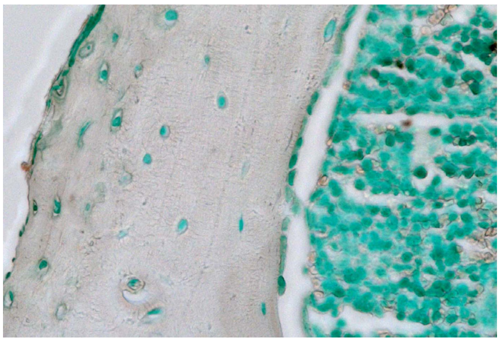

staining with anti- osterix-CON

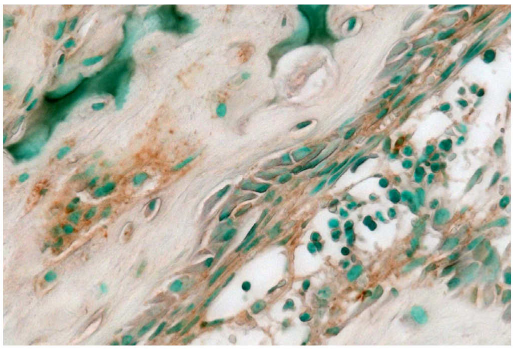

staining with anti- osterix-ΔVHL

Immunocytochemical analysis reveals that the osterix protein is strongly detected in the abundant stromal cells in the diaphyseal regions of the femur.

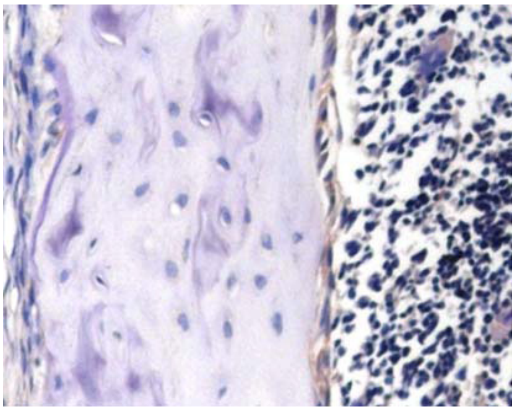

staining with anti- β-catenin-CON

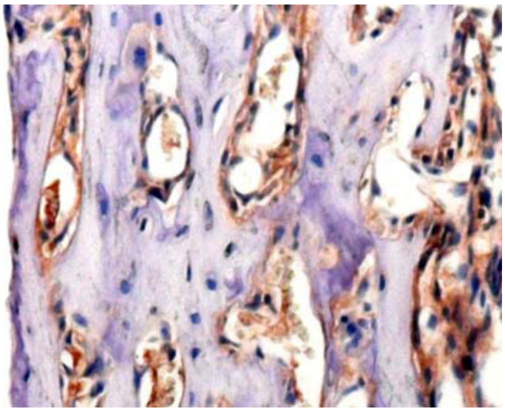

staining with anti- β-catenin-ΔVHL

Sections from 1-month-old CON and ΔVHL mice were IHC-stained using an antibody against β-catenin.

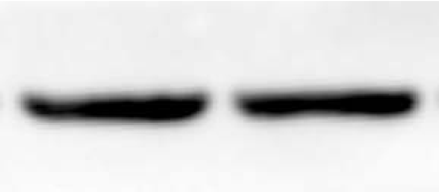

CON-β-catenin -actin ΔVHLβ-catenin –actin

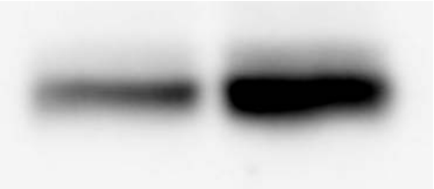

CON-β-catenin ΔVHLβ-catenin

Total protein extracts were prepared from the tibiae of 1-month-old CON and ΔVHL mice and used for western blot analyses for β-catenin.

Raw data for table1

Morphometric data of the osteocytes-Longest / Shortest diameter (um)			
CON-Longest diameter (um)	ΔVhl-Longest diameter (um)	CON-Shortest diameter (um)	ΔVhl-Shortest diameter (um)
12.67	11.17	5.39	3.63
15.74	10.45	4.3	5.13
11.39	12.37	5.16	3.63
9.7	12.03	5.89	5.13
13.13	12.49	5.98	4.38
12.07	9.75	4.28	2.79
12.76	11.58	6.95	3.96
11.39	15.35	6.46	3.08
13.84	12.35	6.15	4.37
14.36	13.85	4.77	4.92
10.12	9.97	4.81	3.11
13.17	10.75	5.99	4.92
14.07	10.73	4.17	4.5
12.85	13.07	4	2.15
12.88	9.02	4.22	3.77
11.77	11.39	4.54	2.98
10.22	9.56	4.17	3.63
11.18	10.13	5.66	2.38
13.85	13.13	5.36	2.98
11.15	13.45	5.74	3.82
12.56	15.51	4.01	4.22
11.47	13.33	6.46	3.48
11.06	13.17		3.21
14.36	10.78		3.04
12.13	10.14		3.64
12.77			3.44
13.44			3.95
12.35			3.97
12.14			2.75
10.86			3.07
12.38			2.44
			3.64
			4.79

			5.19

Morphometric data of the osteocytes-surface area (um^2)/Cell volume (um^3)			
CON-Cell surface area (um^2)	ΔVhl -Cell surface area (um^2)	CON-Cell volume (um^3)	ΔVhl-Cell volume (um3)
663.1	457.98	697.6	474.31
680.1	376.57	635.95	357.57
643.43	430.05	610.5	460.61
699.91	383.3	584.39	352.65
606.06	390.19	705.41	303.11
670.28	374	577.67	432.43
659.15	299.97	639.28	375.68
660.68	329.12	566.66	324.05
627.71	341.24	659.19	363.2
613.65	432.35	620.02	338.43
716.25	331.26	645.91	340.2
600.92	449.53	655.37	446.98
663.04	405.44	619.27	413.49
620.7	439.44	619.4	388.01
684.79	399.56	627.76	305.02
720.91	417.6	605.6	349.64
621.86	361.86	597.76	407.23
	463.73		355.19
			315.81
			279.21
			346.15
			334.21
			450.17
			394.23
			341.52
			411.11
			284.06
			276.81
			286.8

			343.8

Morphometric data of the osteocytes-Nuclear volume(um3)	
CON-Nuclear volume(um3)	ΔVhl-Nuclear volume(um3)
162.77	135.32
158.9	91.91
169	56.74
174.04	147.04
155.72	207.43
172.09	62.4
164.63	75.24
195.18	78.45
185.33	167.77
167.72	208.22
163.55	54.49
153.8	164.49
149.24	176.34
169.95	64.14
151.23	138.22
152.97	53.61
166.88	69.8
173.03	105.89
185.94	86.18
160.9	55.81
193.82	63.83
196.05	67.66
193.11	129.24
198.24	95.57
191	89.65
155.25	95.23
143.74	89.5
183.4	121.22
171.91	161.51
167.87	188.77
195.71	90.66
184.7	53.27
195.87	120.65
162.74	74.86

185.48	158.37
189.09	116.9
173.66	147.78
134.78	184.44
181.58	130.79
182.1	197.14
141.55	151.85
167.95	129.6
196.86	160.57
177.04	69.68
121.78	212.73
171.44	92.4
193.26	67.01
221.76	193.3
166.21	203.88
177.01	191.01
155.42	183.18
191.47	164.82
126.56	152.17
182.24	111.86
155.28	222.7
183.24	162.92
202.94	56.14
185.7	98.48
195.27	114.37
199.86	99.6
182.91	103.15
191.14	71.64
170.54	205.21
161.34	95.9
178.04	94.31
171.04	149.58
180.27	124.69
171.18	170.94
153.12	185.95
159.36	171.07
147.88	160.42
219.09	201.82
131.92	128.35

147.19	100.11
156.1	144.15
195.84	129.32
186.83	211.81
168.43	180.85
162.89	145.59
192.95	161.16
193.34	166.19
191.34	138.44
183.76	115.98
181.15	115.3
150.92	113.33
175.9	203.01
175.28	177.04
156.86	180.55
196.48	179.57
195.05	207.54
141.67	157.03
122.65	146.34
138.9	190.51
159.38	112.75
175.71	123.9
167.5	125.96
137.96	86.99
124.34	223.32
161.53	101.75
185.67	165.95
166.52	158.4
171.31	64.21
195.32	108.12
147.51	101.68
163.11	99.31
	142.4
	164.05
	220.42
	183.57
	167.68
	114.71
	64.3

	94.66
	176.71
	75.16
	141.34
	121.99
	125.87
	133.26
	166.3
	191.05
	121.06
	140.85
	124.92
	139.92
	130.61
	126.83
	116.55
	64.86
	181.76
	86.37
	104.4
	117.98
	85.54
	114.07
	95.9
	138.08
	64.21
	83.33
	79.53
	65.47
	74.91
	69.58
	133.87
	133.23
	141.6
	80.86
	52.08
	59.08
	84.08
	120.18

	121.36
	115.18
	71.3
	81.28
	81.12
	64.51
	87.55
	59.91
	67.62
	49.63

Table 1 Morphometric data of the osteocytes in cortical bone at the femoral diaphysis of ΔVhl

	CON	ΔVhl
Longest diameter (um)	12.38±1.38	11.80±1.75
Shortest diameter (um)	5.20±0.92	3.71±0.84***
Diameter ratio,long/short	2.38	3.18
Cell surface area (um^2)	656.03±37.2	393.51±48.03***
Cell volume (um^3)	627.51±38.07	361.72±31.90***
Nuclear volume(um^3)	171.55±20.5	125.02±46.09***
Volume ratio,cell/nucleus	3.66	2.89

Morphometric data of the osteocytes in cortical bone at the femoral diaphysis of ΔVhl,make table1 as raw data

Raw data for supplement figure 1

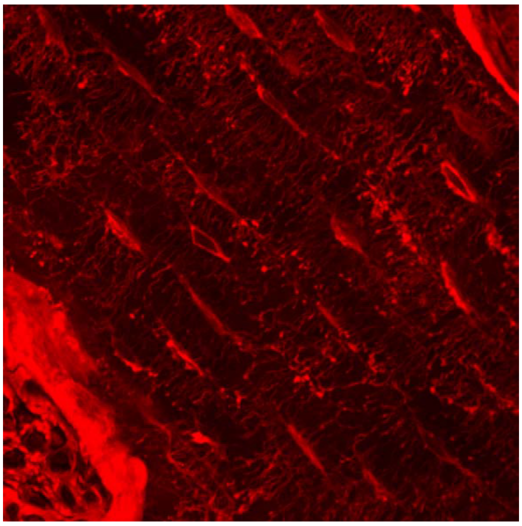

Basic fuchsin staining of parietal bones –CON

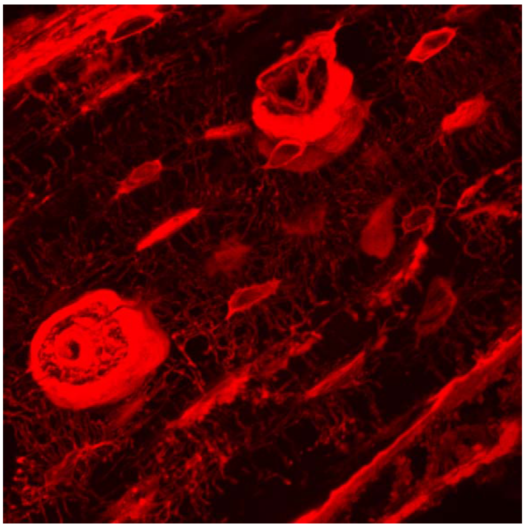

Basic fuchsin staining of parietal bones –ΔVhl

Basic fuchsin staining of parietal bones of the CON and ΔVHL mice (the arrows indicate vascularization in the ΔVHL mouse).

Raw data for rebuttal letter

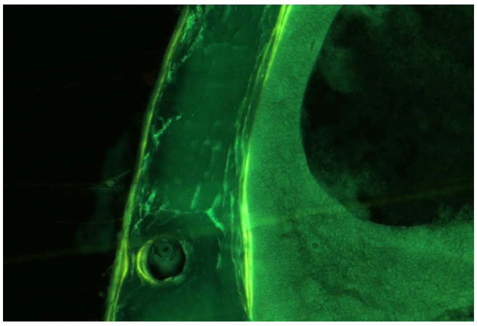

tetracycline double labeling-CON-3W

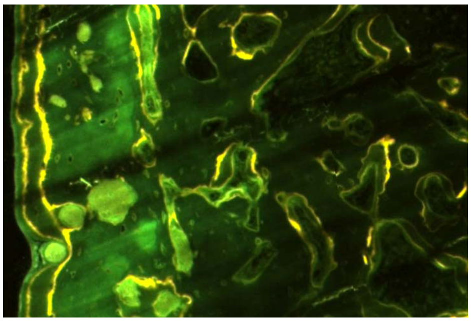

tetracycline double labeling-ΔVHL -3W

Representative images of the histological sections showing tetracycline double labeling in mid-diaphysis of tibia of 3-week-old ΔVHL mice and CON littermates.

CON-Cross-sectional area(mm2)	CON-Cortical bone area(mm2)	CON-Cortical thickness (um)
0.8383754	0.6753035	270.3
0.8825023	0.7213773	275.94
0.8711331	0.7168069	260.61
ΔVhl-Cross-sectional area(mm2)	ΔVhl-Cortical bone area(mm2)	ΔVhl-Cortical thickness (um)
0.9781814	0.8985407	383.22
0.9418332	0.8636025	376.956
0.9827402	0.8908178	398.29
CON-Periosteal surface		
MAR	MS/BS	BFR/BS(um^3/um^2/d)
3.25	68.76	223.47
3.31	65.56	217.0036
3.62	66.67	241.3454
3.43	64.74	222.0582
ΔVhl-Periosteal surface		
MAR(um/d)	MS/BS(％)	BFR/BS(um3/um2/d)
4.825	0.891134	4.299722
4.96	0.845361	4.192998
4.664	0.9123831	4.255355
CON-endocortical surface		
MAR(um/d)	MS/BS(％)	BFR/BS(um^3/um^2/d)
3.65	67.56	246.594
3.71	62.23	230.8733
3.33	68.27	227.3391
3.47	63.15	219.1305
ΔVhl-endocortical surface		
MAR(um/d)	MS/BS(％)	BFR/BS(um3/um2/d)
4.98	96.25	479.325
4.73	93.16	440.6468
4.97	97.46	484.3762
4.96	92.43	458.4528

Static and dynamic histomorphometric measurements (* $p < 0.05$ versus CON mice).

	CON	ΔVhl
Cortical bone		
Cross-sectional area(mm^2)	0.86±0.02	0.97±0.02*
Cortical bone area(mm^2)	0.70±0.03	0.89±0.02*
Cortical thickness (um)	268.95±7.75	386.15±10.97*
Periosteal surface		
MAR(um/d)	3.4±0.16	4.82±0.15*
MS/BS(%)	66.43±1.7	88.30±3.42*
BFR/BS($um^3/um^2/d$)	2.25±0.10	4.25±0.05*
Endocortical surface		
MAR(um/d)	3.54±0.18	4.91±0.12*
MS/BS(%)	65.30±3.05	94.87±2.41*
BFR/BS($um^3/um^2/d$)	2.30±0.10	4.65±0.20*

Static and dynamic histomorphometric measurements,make table as raw data.

Ultimate stress(Mpa)		Ultimate load(N)		Energy to failure(MJ)		Modulus(Mpa)	
CON	ΔVhl	CON	ΔVhl	CON	ΔVhl	CON	ΔVhl
146.6177	171.646	32.38686	96.78407	15.91201	12.93921	5165.871	3427.609
139.5317	188.0202	34.00764	109.943	10.10811	19.0844	4303.325	2824.651
153.2796	204.1198	35.57972	106.8769	9.10551	15.09928	4940.263	3343.626
150.1589	201.374	34.85533	105.4392	6.59807	17.31179	4734.734	3564.81
	202.6151		114.2463		16.76125		3028.708
	229.7237		120.283		17.73527		4233.283

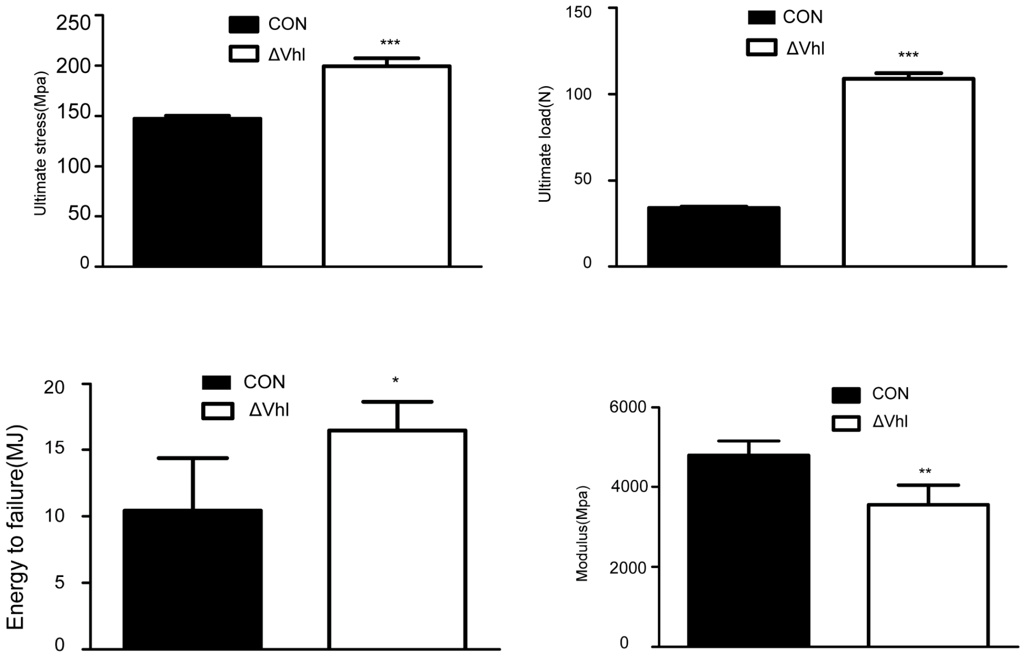

Mechanical parameters of femur measured by three-point bending test from CON and ΔVhl mice,make figure as raw data

Hardness At Max Load (GPa)		Modulus At Max Load (GPa)	
CON	CKO	52.574	42.373
2.79	2.141	44.988	43.231
2.346	1.844	49.26	45.397
2.534	1.594	44.803	37.356
2.16	1.694	39.028	43.152
1.847	2.107	47.253	44.653
2.491	2.065	47.678	30.149
2.369	1.224	52.82	37.933
2.672	1.836	49.125	43.273
2.441	2.181		38.477
	1.934		37.144
	1.848		53.223
	2.925		45.779
	2.081		48.85
	2.172		47.313
	2.3		41.154
	2.095		44.115
	2.144		37.363
	1.823		38.65
	1.919		34.173
	1.579		

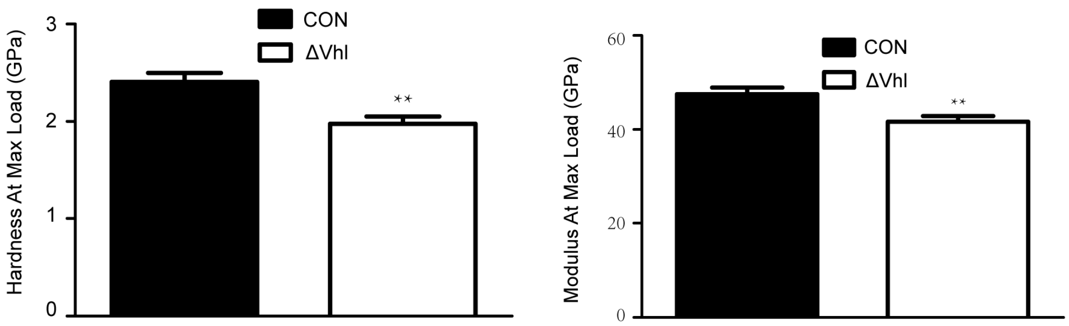

The nanoindentation of mice middle femoral cortical bone at 8-month-old,make figure as raw data

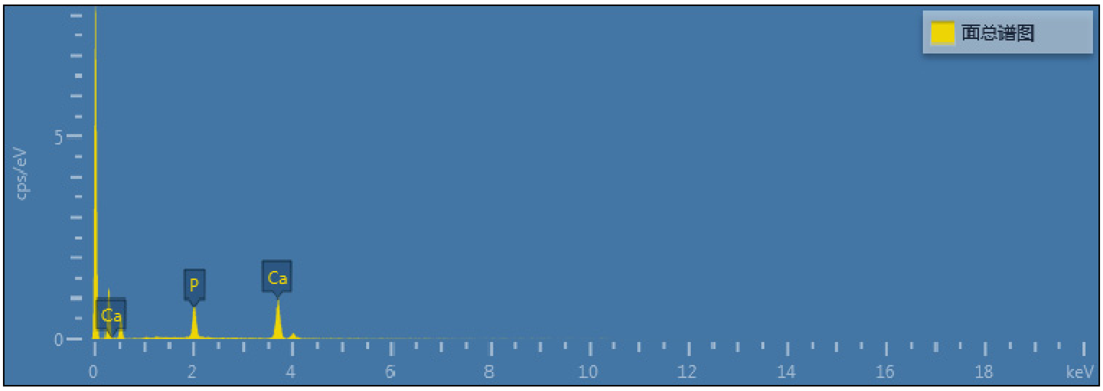

Energy dispersive X-ray spectroscopy (EDX) images-CON

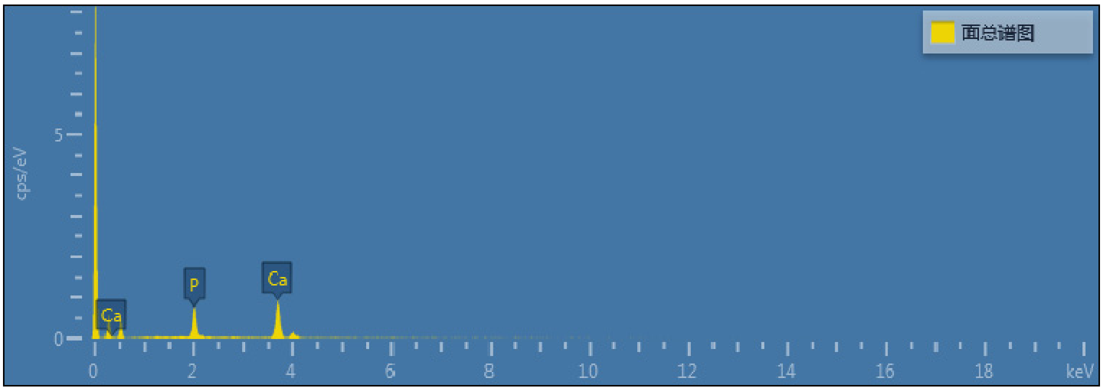

Energy dispersive X-ray spectroscopy (EDX) images-ΔVhl

CON			ΔVHL		
Ca%	P%	Ca/p	Ca%	P%	Ca/p
65.39	34.61	1.88934	66.95	33.05	2.02572
67.18	32.82	2.04692	67.88	32.12	2.11333
68.12	31.88	2.13676	67.79	32.21	2.10463
67.74	32.26	2.09981	67.73	32.27	2.09885
			70.09	29.91	2.34336

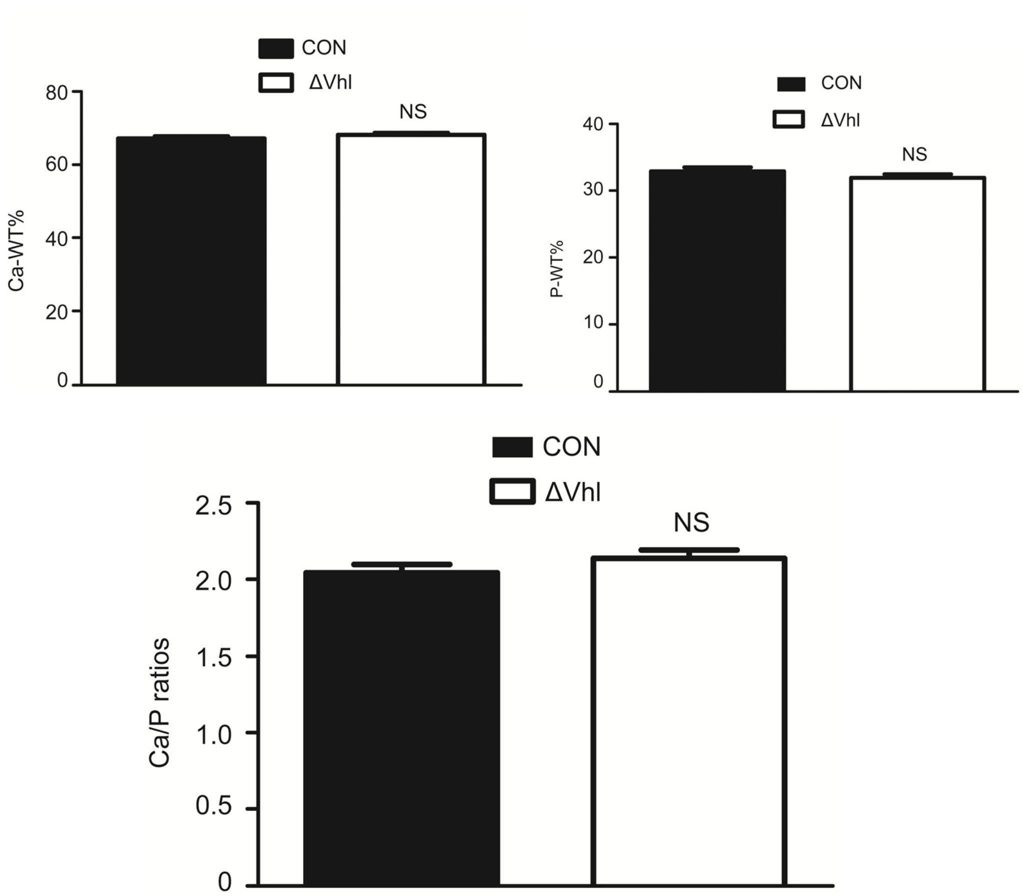

Quantitativef the calcium and phosphorus contents and Ca/P ratios in CON and ΔVhl bone,make figure as table.
